# Ethnomedical Knowledge of Plants Used in Nonconventional Medicine for Wound Healing in Lubumbashi, Haut-Katanga Province, DR Congo

**DOI:** 10.1155/2024/4049263

**Published:** 2024-08-12

**Authors:** Bashige Chiribagula Valentin, Okusa Ndjolo Philippe, Manya Mboni Henry, Bakari Amuri Salvius, Masengu Kabeya Suzanne, Félicien Mushagalusa Kasali, Lumbu Simbi Jean Baptiste

**Affiliations:** ^1^Department of Pharmacology, Laboratory of Therapeutic Chemistry and Analysis of Natural Substances, Faculty of Pharmaceutical Sciences (UNILU), 27, Av. Kato, Commune Kampemba, Lubumbashi, Democratic Republic of the Congo; ^2^Department of Pharmacology, Laboratory of Pharmacognosy, Faculty of Pharmaceutical Sciences, University of Lubumbashi (UNILU), 27, Av. Kato, Commune Kampemba, Lubumbashi, Democratic Republic of the Congo; ^3^Department of Pharmacy, College of Health Sciences, Université Officielle de Bukavu (UOB), PO. Box: 570, Bukavu, Commune of Kadutu, Av. Karhale, Democratic Republic of the Congo; ^4^Department of Chemistry, Faculty of Sciences, University of Lubumbashi (UNILU), 1 Maternity Av., Commune of Lubumbashi, Lubumbashi, Democratic Republic of the Congo

## Abstract

Medicinal plants used for wound healing in Lubumbashi have yet to be discovered. Inventory or profile of their taxa has yet to be established. The present study was carried out to survey the plants used in traditional medicine in Lubumbashi to treat wounds and to define their ethnomedical characteristics. The study was conducted between March 2021 and August 2022, using semistructured interview surveys of households (*n* = 2730), herbalists (*n* = 48), and traditional practitioners: TPs (*n* = 128).The 2,906 interviewed (sex ratio M/F = 0.9; mean age: 56 ± 3 years; and experience: 17 ± 4 years) provided information on 166 taxa, 130 used against chronic wounds, among which *Securidaca longepedunculata* was the top cited. Most of these taxa are shrubs (33%), belonging to 48 botanical families dominated by the Fabaceae (16%). They are indicated in 70 other pathologies. From these 166 taxa, 198 healing recipes are obtained, 11 combining more than one plant. In all these recipes, the leaf (>36%) is the most used part, and the poultice (>36%) is the most popular form of use. Twelve taxa are cited for the first time as medicinal plants, of which *Agelanthus zizyphifolius* has the highest consensus and *Erigeron sumatrensis* has the highest usual value. For the various plants used to treat wounds, some of which are specific to the region, further studies should focus on validating this traditional use.

## 1. Introduction

A wound disrupts routine, typical standard anatomical tissue structure, resulting in loss of epithelial continuity with or without loss of underlying connective tissue and living tissue's anatomical and functional integrity [1, 2]. Different types of wounds, including incised wounds, lacerated wounds, abrasions, contusions, ulcers, and burns, can be distinguished according to their origin, duration, and healing process and grouped into acute and chronic wounds [1, 3]. In response to this structural damage, the body initiates wound healing, a natural process of repair and regeneration of tissue lesions that includes the phases of hemostasis (coagulation), inflammation, proliferation (angiogenesis, granulation, and reepithelialization), and tissue remodeling [4, 5].

Wounds that have not followed the normal healing process and remain open for more than a month or whose size has not contracted by more than 50% during this period are considered chronic wounds [6–8]. Based on etiology, the Wound Healing Society classifies chronic wounds into four categories: pressure ulcers, diabetic ulcers, venous ulcers, and arterial insufficiency ulcers [9]. Chronic wounds cause severe emotional and physical trauma for not only the patient but also their family [10, 11]. Chronic wounds can also lead to disability after all available therapeutic interventions have been exhausted and amputation deemed necessary [10, 12]. The worldwide prevalence of chronic wounds is estimated at 1.51 and 2.21 per 1000 inhabitants [13], and 1 to 2% of the population in developed countries has already been diagnosed with chronic wounds [14]. This rate is three times higher in DRC, where access to primary healthcare and hospital technical facilities suffers enormous shortcomings. Incidence is set to rise as populations age worldwide [15], and the risk of developing a chronic wound is 1.72 times higher in men than in women [16]. Chronic wounds are, therefore, a significant health problem with various consequences, affecting patients' physical, social, and mental health as well as the costs of healthcare systems. Their prolonged healing time, economic burden, reduced quality of life, increased risk of infection, and impact on patient mobility and functionality make them a significant concern for healthcare professionals [17]. In Lubumbashi, there are many cases of chronic wounds, due to the high level of mining activity and other physical activities, combined with poor access to adequate care, which favors cases of infected wounds [18].

The current therapeutic arsenal offered by conventional medicine is varied, ranging from regenerative medicine and stem cell therapies to biological dressings and scaffolds, negative pressure wound therapy (NPWT), electrical stimulation, topical growth factors and cytokines, hyperbaric oxygen therapy (HBOT), advanced dressings, and artificial intelligence [9, 17]. Despite the existence of these treatment methods, chronic wounds are among the pathologies for which the people of Lubumbashi most often turn to nonconventional medicine as in many cases biomedical treatment has resulted in therapeutic failure on the one hand, but on the other, the population has more confidence in alternative medicine due to sociocultural habits [18, 19]. In addition to the abovementioned reasons, it should be remembered that the DRC, particularly the city of Lubumbashi, suffers from poor health coverage. The low rate of access to primary health care could also justify the high demand for nonconventional medicine [18]. This nonconventional medicine remains dominated mainly by medicinal plants and is poorly documented in Lubumbashi [20].

The ethnomedical use of plants in wound treatment is not only cheap and accessible but also a reliable natural resource of medicinal substances. Studies on medicinal plants have confirmed that herbal medicines have fewer side effects than chemical agents and are more cost-effective [21–23]. As medicinal plants constitute an essential resource of substances with beneficial therapeutic effects, in various African countries, plants with wound-healing potential have been investigated from both the ethnobotanical and ethnopharmacological points of view [24–27].

A link between ethnopharmacological activity and bioactive groups has justified the traditional use of some medicinal taxa for wound treatment [28–31], and several phytomedicines have been developed, such as Cicaplast® used for the treatment of acute wounds [32] and Cicatryl® used for the treatment of superficial wounds [33].

In DRC, to our best knowledge, a single ethnobotanical survey was carried out on wound-healing medicinal plants. It reported 53 taxa used as healing plants in Congolese pharmacopeias [34]. Examination of this study shows that the taxa described tend to come from the western part of the DRC, which is not representative of the whole country due to the climatic variability that characterizes each region of the country. This is particularly true of Haut-Katanga Province, where the Miombo Forest is characterized by other types of vegetation, and the population living there has different cultural practices to those found in the west of the country.

The present study aims to inventory the plants and their healing recipes used in nonconventional medicine in Lubumbashi and to draw up their ethnomedicinal characteristics. Aware that the general population practices this medicine, traditional healers and herbalists, we have focused the study on three population categories (households, herbalists, and traditional healers) to gather as much information as possible about wound care in the region.

## 2. Materials and Methods

### 2.1. Experimental Framework

This study was conducted in the seven communes of Lubumbashi City: Annexe, Kampemba, Katuba, Kamalondo, Kenya, Lubumbashi, and Rwashi, in the province of Haut-Katanga in the Democratic Republic of Congo.

**Figure 1:**
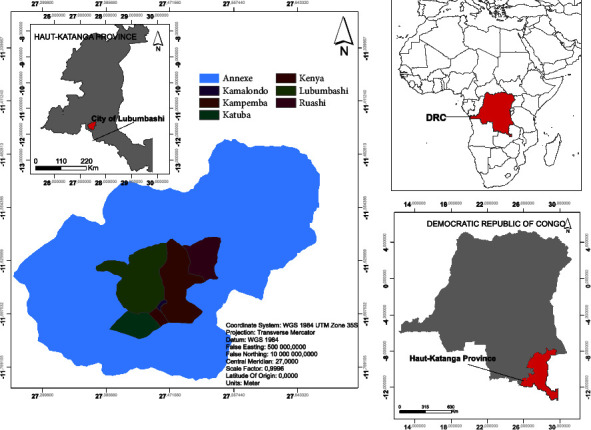
Map of the city of Lubumbashi in Haut-Katanga, DRC.

Lubumbashi is located between 11°26′–11°55′ north latitude and 27°15′–27°40′ east longitude at an altitude of 1,230 meters. The climate is tropical, with an average annual temperature of 22.4°C and an average annual rainfall of 512.7 mm^3^. It has two seasons, with a shorter rainy season (November to April). The Miombo clear forest dominates typical vegetation [35] (Figure 1).

### 2.2. Ethnomedical Data Collection

Ethnobotanical data were collected from households, herbalists, and traditional practitioners (TPs) in Lubumbashi between March 2021 and August 2022. The sample size of household respondents was determined using two-stage sampling: a stratified sample and a cluster sample. For each commune considered a stratum, the corresponding commune office provided the population size. The size of the representative sample at this level was determined by the following formula [36]:

(1) $$\begin{array}{c} n=\frac{t_{p}^{2}*p\left(1-p\right)*N}{t_{p}^{2}*p\left(1-p\right)+\left(N-1\right)*y^{2}}, \end{array}$$

where *n* is the sample size, *N* is the actual population size, *p* is the expected proportion of respondents (we estimated *p* at 0.5), *t*_*p*_ is the sampling confidence interval (*t*_*p*_ = 1.96 given that we set a 95% confidence interval), and *y* is the margin of sampling error: we put it at 0.05.

For this study, given that for a population of over 20,000 households, the sample size varies little for margins of error greater than 5%, and we adjusted the minimum size with an excess of over 1.5% (Table 1).

The sample determined by commune was divided by avenue, considered clusters by counting every three households. In each household, we included any adult (≥18 years old) who had ever used plants to treat wounds, provided they were too unfamiliar with the interviewers.

Due to a lack of reliable data on the size of the herbalist population in Lubumbashi, we aimed to interview as many herbalists as possible, accessible through markets and other public spaces. This stage enabled us to meet 48 herbalists who agreed to participate in the study.

Respondents in the traditional practitioner category were determined from a list of 50 TPs drawn up during a recent survey in the study region [35]. This stage resulted in 45 TPs, all of whom agreed to participate. From them, snowball sampling was carried out to increase the sample size, which enabled us to reach a further 85 TPs, 83 of whom agreed to participate in the study and 4 of whom were on the initial list. The TP survey was thus carried out on 128 respondents.

### 2.3. Data Processing and Analysis

During the surveys, the plants were identified in the local languages. The specimens were collected in the company of the informants, and herbaria were compiled and deposited at the Kipopo Herbarium for the attribution of scientific names. Subsequently, these records were updated through comparison with the following databases: the African Plant Database (https://africanplantdatabase.ch/) Plants of the World Online (https://powo.science.kew.org/) and the World Flora Online (http://www.worldfloraonline.org/).

Three ethnomedicinal indexes were determined to assess significant species (data): the therapeutic consensus index (TCI), the medicinal usage index (MUI), and the medicinal capability index (MCI). The plant therapeutic consensus index (TCI) was calculated using the formula:

(2) $$\begin{array}{c} \text{TCI}=\frac{Np}{N}, \end{array}$$

where Np is the number of people who mentioned the plant and *N* is the number of people surveyed during the study.

The medicinal use index (MUI) was calculated using the formula:

(3) $$\begin{array}{c} \text{MUI}=\frac{\text{NUi}}{\sum \text{Up}}, \end{array}$$

where NUi is the number of uses of plant *i* and ∑up is the set of uses of all plants inventoried during this study. The medicinal capability index (MCI) was calculated by the formula:

(4) $$\begin{array}{c} \text{MCI}=\frac{\text{nt}}{\text{Nt}}, \end{array}$$

where nt is the number of taxa cited in the management of a given pathology and Nt is the total number of taxa inventoried during the study.

In addition to the three ethnomedicinal indexes mentioned above, the relative citation frequency (RCF) was also determined. It was calculated using the formula:

(5) $$\begin{array}{c} \text{RCF}=\frac{n}{N}\times 100, \end{array}$$

where *n* is the number of occurrences of the factor examined and *N* is the total number of the population concerned. This parameter was used to quantify various factors analyzed in this study, except for those for which ethnobotanical indexes were applied.

In this study, the MUI highlights the level of medicinal use of a plant among the population studied; TCI enables us to identify the level of consensus among the population on the healing use of a given species and, hence, its importance in managing wounds in the region. As this is a targeted ethnobotanical study, the medicinal consensus index corresponds to citation frequency. MCI enables us to measure the potential of a given community to manage a well-defined pathology.

## 3. Results

### 3.1. General Characteristics of Inventoried Plants

**Figure 2:**
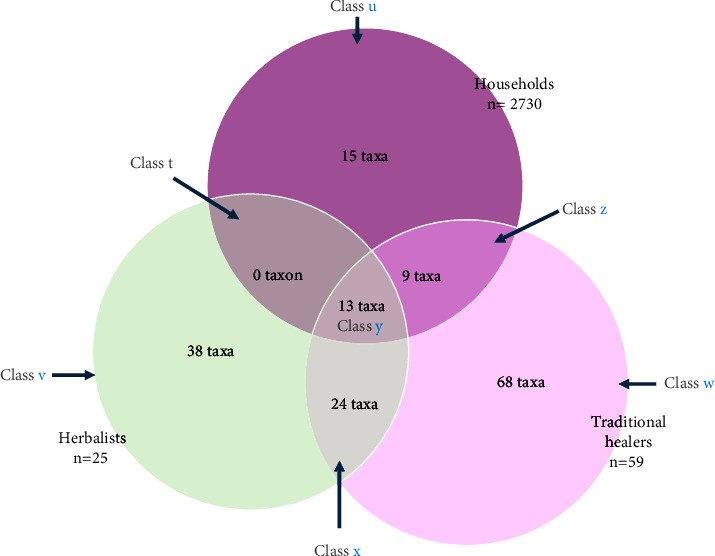
Distribution of taxa by informant source.

This study identified one hundred and sixty-six plant species used as healing plants in Lubumbashi, 130 of which are used to treat chronic wounds. These plants were collected from households, traditional practitioners, and herbalists. Depending on the origin of the information obtained for each taxon during the surveys, we can group the plants inventoried during this study into 6 classes: *u*, *w*, *v*, *x*, *z,* and *y* (Figure 2).

- Class *u*, made up of 15 taxa from households alone, where *Securidaca longepedunculata, Musa × paradisiaca, Manihot esculenta, Jatropha curcas, and Tetradenia riparia *occupy the top five positions according to their TCI: therapeutic consensus index (0.306–0.689)
- Class *w*, comprising 68 taxa from traditional practitioners alone, where *Sterculia quinqueloba, Ziziphus abyssinica*, and *Triumfetta rhomboidea* occupy the top 3 positions in terms of TCI (0.027–0.043)
- Class *v*, comprising 38 taxa from herbalists alone, with *Annona senegalensis*, *Dysphania ambrosioides*, *Albizia antunesiana*, *Chenopodium opulifolium*, and *Crossopteryx febrifuga* in the top 5, with MCIs (medicinal capability indexes) ranging from 0.014 to 0.017
- Class *x*, comprising 24 taxa from both traditional practitioners and herbalists, where *Sterculia quinqueloba*, *Ziziphus abyssinica*, *Triumfetta rhomboidea*, *Uapaca sansibarica,* and *Terminalia mollis* occupy the top 5 positions (TCI: 0.013–0.024)
- Class *z*, comprising 9 taxa from both traditional practitioners and households, where the top 5 positions are occupied by *Acalypha cupricola*, *Acalypha homblei*, *Acalypha ornata*, *Acalypha chirindica*, and *Vachellia karroo*, with a TCI varying between 0.046 and 0.081
- Class *y*, comprising 13 taxa common to all 3 sources, of which the top 5 according to their MCI values (0.048–0.087) include *Allium sativum*, *Bobgunnia madagascariensis*, *Ficus sur*, *Euphorbia hirta*, and *Cassia abbreviata* (Table 2)

**Figure 3:**
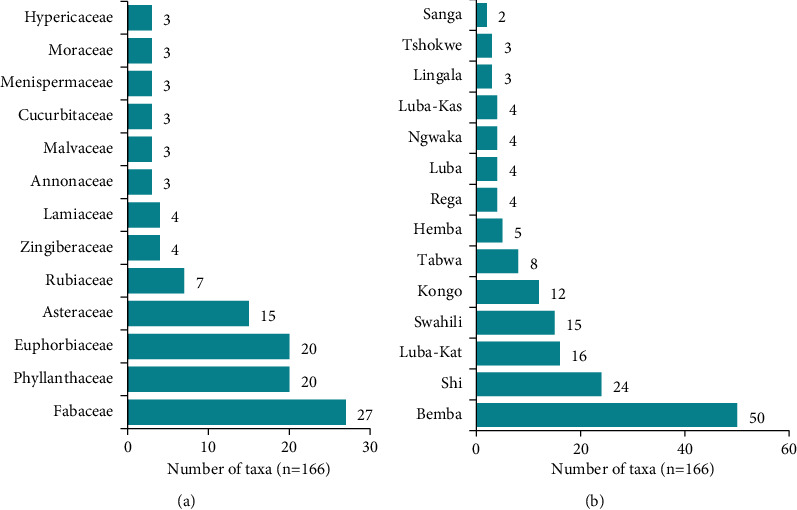
Families with *n* ≥ 3 taxa (a) and plant naming languages with *n* ≥ 2 taxa (b).

These taxa belong to 48 families (Table 2), only 13 of which contain at least 3 taxa among those inventoried during this study. These families are dominated by the Fabaceae (27 taxa), followed by the Phyllanthaceae and Euphorbiaceae with 20 taxa each. The Asteraceae family ranks fourth with 15 taxa, followed by the Rubiaceae with 7 (Figure 3(a)). Each of these inventoried plants is named in one of the languages of the 26 Congolese ethnic groups, of which Bemba (31%), Shi (14%), Luba-Kat (12%), Swahili, and Kikongo, with an RCF of over 8% each, occupy the top five places (Figure 3(b)).

Nine plants are the most widely used in the study area, with the medicinal usual index: MUI >0.090. These are, in order of precedence, *Lantana camara* (0.194), *Gardenia ternifolia* (0.181), *Tetradenia riparia* (0.139), *Xylopia aethiopica* (0.139), *Euphorbia hirta* (0.125), *Garcinia huillensis* (0.111), *Cucumis melo* (0.111), *Curcuma longa* (0.097), and *Nauclea latifolia* (0.097) (Table 2).

**Figure 4:**
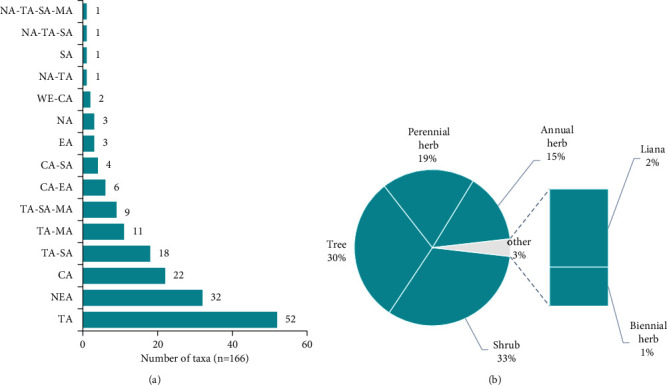
Geographical (a) and morphological (b) types. TA: intertropical Africa; NEA: Northeast Africa; CA: Central Africa; SA: southern Africa; MA: Madagascar; EA: East Africa; NA: North Africa; WE: West Africa.

These plant taxa comprise 7 morphological types, dominated by the shrub type (33%), followed by the tree type, which represents 27% of taxa (Figure 3), and also include 15 geographical types, dominated by the intertropical African type, which shall consist of 32% of taxa (Figure 4).

Of the 166 taxa listed, *Securidaca longepedunculata* (0.689), *Musa* × *paradisiaca* (0.642), *Manihot esculenta* (0.353), *Jatropha curcas* (0.314), and *Tetradenia riparia* (0.306), with a therapeutic consensus index: TCI >0.3, are the most consensual and most cited as healing plants for this study. They all come from class *w*, a class dedicated to plants sourced solely from resource persons encountered in households (Table 2).

The literature consulted in relation to the 166 taxa inventoried during the surveys shows that one hundred and eighteen taxa (71%) have never been studied from the point of view of assessing their healing activity. Of these, 70 are reported for the first time as plants used in wound care, including 12 taxa reported for the first time as medicinal plants, of which *Agelanthus zizyphifolius* (MCI = 0.042) has the highest consensus factor and *Erigeron sumatrensis* (5 uses) has the highest usual value. These taxa are used in the management of chronic wounds (Table 2).

### 3.2. Ethnomedical Profile of Inventoried Plants

**Figure 5:**
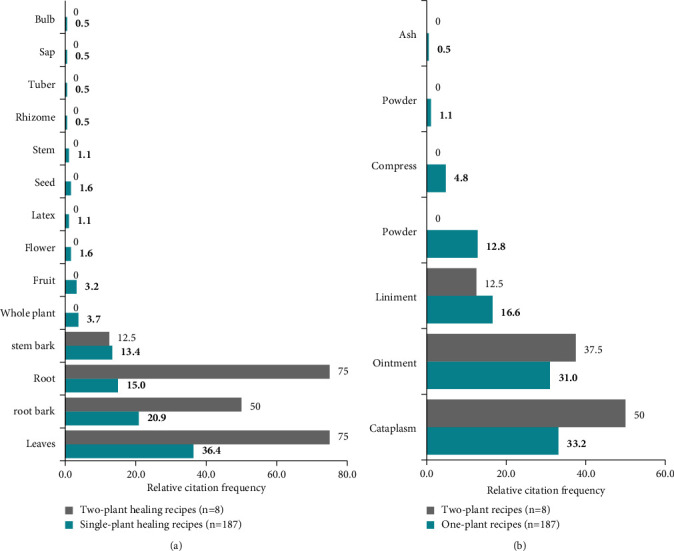
Parts used to treat wounds (a) and types of use for healing recipes (b). These data are expressed in terms of relative citation frequency.

The 166 plants identified in this study during the surveys are used in 198 healing recipes, of which 187 use a single plant (Table 2) and 11 combine two plants (Table 3). In these essentially topical recipes, the leaf is the organ most frequently used (36.9% in monophytotherapy and 75% in biphytotherapy). Root barks follow it in monophytotherapy and roots in biphytotherapy. Cataplasm is the most frequently used form of administration (36.4% for monophytotherapy and 50% for biphytotherapy), followed by ointment (Figure 5).

In monophytotherapy, recipes based on *Securidaca longepedunculata* (root bark), *Musa* × *paradisiaca* (leaves), *Manihot esculenta* (leaves), *Jatropha curcas* (leaves), and *Tetradenia riparia* (leaves) are the most widely accepted, with TCI >0.3 (Table 2). In the polyherbal recipe, the recipe based on a mixture of *Moringa oleifera* (leaves) and *Garcinia guillen* (root bark) with a TCI of 0.33 was the most widely cited. The taxa, *Ageratum conyzoides*, *Euphorbia hirta, Garcinia huillensis*, and *Moringa oleifera,* are each used in 2 different recipes for plants used in mixtures (Table 3).

The taxa inventoried in this study are involved in the management of 70 other pathologies, of which fever, malaria, DTIs (digestive tract infections), diabetes, and STIs (sexually transmitted infectious diseases), with a RCF ≥40% of subjects consulted, are the most cited. However, diabetes, DTI, malaria, STIs, diarrhea, fever, and dysentery are the conditions with the highest potential for medicinal treatment in the study region, MCI: 0.102–0.259 (Table S1).

### 3.3. Sociodemographic Profile of Interviewed Subjects

The persons consulted in this study were either household contacts (93.9%), herbalists (1.7%), or practitioners of traditional medicine (4.4%), primarily women (56.4%), most of whom were between 50 and 60 years old (extremes: 18 and 71). The majority had a secondary education (39.0%). These persons were found in the 44 districts that make up the seven communes of the city of Lubumbashi in similar proportions (13.9–15.1%). They were engaged in 6 occupation types, the most representative of which was housework (52.6%). In most cases, they have more than 11 years of experience using medicinal plants (Table 4).

## 4. Discussion

Katangese flora is rich in plant taxa, the ethnomedical knowledge of which needs to be better reported. Through this study, we propose to report on ethnomedical knowledge of plants used in wound care within households and among herbalists and traditional practitioners in the region.

### 4.1. General Characteristics of Inventoried Plants

This study reports on 166 taxa used in nonconventional medicine in Lubumbashi City to treat wounds, both directly by the general population (*n* = 37 taxa) and via traditional medical practitioners (*n* = 114 taxa) and herbalists (*n* = 75). Of the 166 taxa inventoried, 15 (i.e., 9% of all taxa) are known only to the household population. Of these 15 taxa, only *Uapaca kirkiana and Cucumis sativus* have no scientific proof of their healing properties. The fact that over 80% (13 taxa) of these plants have pharmacological evidence of healing activity (Table 2) shows that the general population has a proven knowledge of wound care, and it is likely that the two taxa not yet studied are probably active.

This result also shows that traditional medical practitioners strongly influence the region's ethnomedical knowledge, which is also available to the general population. It should be remembered that 15 taxa are known only to households. This suggests that traditional medicine professionals hold specific knowledge in Lubumbashi. There are two possible reasons for this: on the one hand, it must be recognized that in this region, medical knowledge is acquired through several channels, including dreams and family [19, 35, 39, 54, 113, 125–127], which the families of professional healers do not monopolize; on the other hand, today's globalization has given free access to written information from different cultures, which may constitute another source of knowledge that does not fall within the monopoly of the practice of traditional medicine. However, the fact that many taxa—68 or 41% (Figure 2)—were found only among traditional healers shows that they remain masters of their art, possessing necessary knowledge not shared with any other category.

The study also shows there are common plants between traditional practitioners and households, whereas there are none between herbalists and the general population (Figure 2). This lack of intersection between the two categories may reflect the need for more collaboration between herbalists and households regarding knowledge sharing. This could be justified by the fact that herbalists would lose out if the general population came to know their recipes, especially as this population is the herbalists' target group. In this context, commercial interests would take precedence over human interaction. Moreover, the cross-fertilization of knowledge between TPs and households may be because, in most cases in the study region, the acquisition of TP knowledge emanates from ancestors [35, 54, 128]. As a result, ethnomedical knowledge constitutes a family heritage that can be shared between members of the same family without these members practicing traditional medicine as their professional activity. As a result, they would be more open to sharing their knowledge with friends, and knowledge could easily pass from one family to another.

This study shows that more than a third of the plants are shrubs or trees (Figure 4), endemic to intertropical Africa (Figure 4), belonging to several families with a predominance of Fabaceae. These results are in line with the literature. Indeed, the Fabaceae is the most crucial tree family in African tropical and dry forests [129]. This importance of the Fabaceae is observed both in the plant kingdom [130] and in the category of African medicinal plants [131]. The numerical predominance of Fabaceae in sub-Saharan Africa has been attributed to their ability to capture atmospheric nitrogen, enabling them to grow in nutrient-rich and nutrient-poor soils [131]. In our environment, no accessible study has addressed the question of the preponderance of one botanical family over all the taxa used in traditional medicine in the region.

However, analysis of accessible ethnobotanical studies in Katanga to inventory medicinal plants shows that the Fabaceae family is the most frequently mentioned. Except for a survey carried out by the OCU (*Observatoire de Changement Urbain*) equal to observatory of urban changes, under the auspices of the University Cooperation for Development (UCD) [19] about healers and medicinal plants in Lubumbashi, which inventoried 132 taxa for which Fabaceae, with 14 taxa, occupies first place, the other studies targeted a specific ethnomedical use. These include the study of plants used to treat gastrointestinal disorders in Kamina and Kanyama: *n* = 10 taxa, Fabaceae = 2 taxa [132]; the survey of anticariogenic plants in Lubumbashi: *n* = 14 taxa, Fabaceae = 3 taxa [113]; the study of antimalarial plants in the commune of Kenya (Lubumbashi): *n* = 13, Fabaceae: 6 taxa [126], from the whole city of Lubumbashi: *n* = 19, Fabaceae: 11 taxa [127], and from Lubumbashi and its surroundings: *n* = 96, Fabaceae = 22 taxa [54]; the study on antidiabetic plants from Lubumbashi and its environs: *n* = 45 taxa, Fabaceae = 11 taxa; the survey of plants used against urogenital schistosomiasis: *n* = 61, Fabaceae = 17 taxa; the study on plants used in the management of sexual dysfunctions in the Kampemba commune (Lubumbashi): *n* = 21, Fabaceae = 7 taxa [47]; the study on plants used in Lubumbashi and surrounding areas to treat gastritis: *n* = 14, Fabaceae = 3 taxa [133]; and the survey of plants used in Lubumbashi to treat typhoid fever: *n* = 54, Fabaceae = 20 taxa [35].

It should be noted, however, that ethnomedical studies targeting healing plants in other regions place other families, notably the Asteraceae, first. This is the case of the survey on Iranian healing plants: *n* = 20, Asteraceae = 3 taxa [134]. This was also the case for a study that inventoried African healing plants: *n* = 61 taxa, Asteraceae = 5 taxa [135], most of which were drawn from West African pharmacopeias. The same applies to the study that reported 14 of the most widely used healing plants in the Northern Hemisphere: Asteraceae = 5 taxa [28]. This disparity can be justified because each region has its floristic characteristics. The Fabaceae family predominates among woody taxa in our study region, where the Miombo clear forest is predominant [136, 137]. It would nevertheless be desirable to carry out a generalized ethnomedicinal study to determine unequivocally the dominant family in the category of medicinal plants in the region.

The literature review on the 166 taxa inventoried during this study shows that 17 taxa are reported among the 53 recently mentioned as healing plants in DRC [77]. Analysis of the data collected during our research about the literature available on these 166 taxa inventoried enables us to group these plants into 3 classes: A, B, and C (Table 2).

Class A would comprise the 45 taxa for which the literature contains pharmacological evidence of healing activity. The most widely accepted taxa in this class are *Securidaca longepedunculata*, *Musa* × *paradisiaca*, *Manihot esculenta*, *Jatropha curcas*, and *Tetradenia riparia*, with a TCI >0.3 (Table 2).
Class B would comprise taxa reported in the literature to be used as healing plants in nonconventional medicine without any pharmacological studies validating this use. This class would comprise 48 taxa, which can also be subdivided into three subclasses: (iia) subclass B1 includes 22 taxa reported as healing plants in other Congolese ethnobotanical studies. In this subclass, we find *Uapaca nitida, Acalypha homblei*, *Aframomum angustifolium*, *Baphia capparidifolia*, *Dysphania ambrosioides*, *Bridelia duvigneaudii*, *Caladium bicolor*, *Combretum racemosum*, *Albizia gummifera*, *Khaya nyasica*, *Uapaca kirkiana*, *Memecylon flavovirens*, *Hymenocardia acida*, *Imperata cylindrica*, *Euphorbia hypericifolia*, *Penianthus longifolius*, *Euphorbia tirucalli*, *Shirakiopsis elliptica*, *Euphorbia inaequilatera*, *Maprounea africana*, *Uapaca sansibarica, Uapaca pilosa,* and *Zanthoxylum chalybeum*; (iib) subclass B2 comprises 6 taxa reported in both traditional Congolese and non-Congolese medicine. In this subclass, we find the *taxa Annona senegalensis*, *Crossopteryx febrifuga*, *Bridelia atroviridis*, *Capsicum frutescens*, *Pseudolachnostylis maprouneifolia,* and *Gymnanthemum amygdalinum*; and (iic) finally, subclass B3 is made up of 20 taxa that are only reported as healing plants outside the DRC. In this subclass, we find *Cassia abbreviata*, *Celosia trigyna*, *Commelina diffusa*, *Acalypha ornata*, *Vachellia karroo, Dichrostachys cinerea*, *Croton mubango*, *Entada africana*, *Carduus nyassanus*, *Albizia adianthifolia*, *Combretum molle*, *Ageratum conyzoides*, *Parinari curatellifolia*, *Eleusine indica*, *Flueggea virosa*, *Ochna schweinfurthiana*, *Psorospermum febrifugum*, *Syzygium guineense*, *Xylopia aethiopica,* and *Ziziphus mucronata* (Table 2).
Class C would include 70 taxa for which no previous ethnopharmacological or ethnomedical data relating to wound healing have been reported. These 70 taxa are, therefore, reported for the first time as healing plants. Thus, this class reflects the specificity of the population of Lubumbashi in the management of wounds in nonconventional medicine. Based on literature data, the taxa in this class can be subdivided into the following 4 subclasses:
    (iiia) Subclass C1 includes 28 taxa that are reported as medicinal plants in the region in previous research. In this subclass, we find *Anisophyllea boehmii*, *Antidesma venosum*, *Cucumis sativus*, *Dalbergia boehmii*, *Droogmansia munamensis*, *Diplorhynchus condylocarpon*, *Entada abyssinica*, *Mucuna poggei,* and *Terminalia mollis*, cited in the cohort of antidiabetic plants in the region [128]; *Acalypha chirindica*, *Acalypha cupricola*, *Acalypha paniculata*, *Acalypha psilostachya*, *Antidesma membranaceum*, *Bridelia scleroneura*, *Oldfieldia dactylophylla,* and *Phyllanthus parvulus*, cited in the area in antimitotic plant's cohort [39, 138]; *Dalbergia nitidula*, *Justicia insularis* [54], *Ekebergia benguelensis,* and *Landolphia congolensis* [127] cited in the region's antimalarial plant; *Aframomum alboviolaceum*, *Garcinia huillensis*, and *Monotes katangensis*, cited in the cohort of plants used against urogenital schistosomiasis [125]; *Senna petersiana* [139], cited as a plant used against erectile dysfunction; *Bobgunnia madagascariensis*, cited in the cohort of anticariogenic plants [113]; *Albizia antunesiana* [35], reported in the cohort of antityphoidal plants; and *Aloe buettneri*, registered in the cohort of plants used against gastrointestinal disorders in cattle [140].
    (iiib) Subclass C2 comprises 14 taxa reported as medicinal plants in other regions of the DRC outside Katanga. In this subclass, we find *Sterculia quinqueloba*, registered in the cohort of antisickle cell anemia plants [141]; *Heinsia crinita* [142], *Chenopodium opulifolium*, *Crassocephalum montuosum*, *Hypoestes triflora*, *Jacobaea maritima*, *Psorospermum corymbiferum* [143], *Albertisia villosa*, *Anonidium mannii* [144], *Cyanthillium cinereum*, *Kalaharia uncinata*, *Steganotaenia araliacea, and Zanha africana* [145], reported as antimalarial plants in DRC; and *Mitragyna stipulosa* [52], reported in the country's antidiabetic plant cohort. This study shows that these plants are also known and used in traditional medicine in Lubumbashi, as in other regions of the country. This reflects the cultural diversity of conventional medicine of Lubumbashi and can be compared with the disparity observed in the languages used to name the plants (Figure 3(b)), where we find the languages of ethnic groups not native to the region, which also shows the cultural mixing of knowledge.
    (iiic) Subclass C3 comprises 16 taxa reported as medicinal plants in several African countries outside the DRC. These include *Acalypha petiolaris* reported in Zimbabwe [50]; B*idens grantii* in Kenya [146] and Uganda [147]; *Plectranthus esculentus* [syn *Coleus esculentus*] [148], *Smilax anceps* [149], *Senna siamea* [150], *Triumfetta rhomboidea* [151], *Uapaca acuminata* [152] in Nigeria, and *Hibiscus surattensis* [153] in Rwanda; *Distephanus biafrae* [syn *Vernonia biafrae*] [154], *Nicandra physalodes* [155], and *Phyllanthus ovalifolius* in Ethiopia [156]; *Khaya anthotheca* in Cameroon [157]; *Luffa aegyptiaca* in Egypt [158]; and *Markhamia lutea* [159] and *Senna singueana* in Tanzania [160].
    (iiid) Subclass C4 contains taxa not reported in any previously accessible scientific literature on medicinal use. In this group, we find the taxa *Agelanthus zizyphifolius*, *Cleistanthus polystachyus*, *Eriosema glomeratum*, *Erigeron sumatrensis*, *Euphorbia terracina*, *Ficus ampelos*, *Gardenia imperialis*, *Monotes africanus*, *Polhillides velutina*, *Vernonia excelsa*, *Uapaca acuminata,* and *Uapaca robynsii*. The ethnobotanical information in this study for these taxa represents their first reported ethnobotanical uses. These taxa aligned with the list of medicinal taxa specific to the region.

The fact that 45 taxa (27%) inventoried in this study have pharmacological evidence as healing plants (class A) lends credibility to the information gathered from the study's resource persons and suggests a high probability of finding taxa with proven healing activity among class C plants [35, 161].

The existence of class B taxa, i.e., those with the same wound-healing use in our study environment as elsewhere, according to the available literature, may constitute another factor of credibility for the information gathered from resource persons during this study, especially significant as this fact reinforces the consensus around the use of these taxa as healing plants. The existence of class B may also reflect cultural interference with traditional medicine from Lubumbashi. This interference may be justified not only by the migratory movements experienced by the city as the country's economic province but also by the evolution of new information technologies, which facilitate access to knowledge from a distant region without the physical cultural cross-fertilization necessarily having been observed.

The existence of class C taxa shows that, despite the cultural interference to which the traditional medicine of Lubumbashi is subject, it nonetheless has several distinctive features. Class C taxa are critical targets for further biological screening to validate the healing uses of plant resources specific to the region. Together with the taxa in class B, they represent a possible extension of this ethnomedical study into ethnopharmacological studies. This work is in progress in our laboratory.

Although taxa in class C and particularly in subclass C4 are not reported in the literature as healing plants or, even better, as medicinal plants, some taxa in the same genera are reported as healing plants. For example, in the genus Agelanthus, the methanolic extract of the leaves of *Agelanthus dodoneifolius* (DC) Polhill and Wiens from Nigeria is reported to have antiulcer activity at 800 and 1600 mg/kg body weight on the *Rattus norvegicus L.* model when administered orally [162]. In the Cleistanthus genus, *Cleistanthus collinus* (Roxb.) Benth. ex Hook. f. is reported to be a healing plant used in India [163]. In the genus Eriosema, three taxa, *Eriosema chinense* Vogel (seed), *Eriosema cordatum* E. Mey. (roots), *Eriosema montanum* Baker f. (leaves), and *Eriosema psoraloides* (Lam.) G. Don (leaves and roots), are reported as healing plants in various African countries [164].

In the genus Erigeron, *Erigeron floribundus* (Kunth) Sch. Bip. (leaves, roots, and flowers) [165] is reported as a healing plant. In the Euphorbia genus, the aerial parts of 5 taxa, *Euphorbia characias L.*, *Euphorbia helioscopia L.*, *Euphorbia macroclada* Boiss., *Euphorbia seguieriana* Neck, and *Euphorbia virgata* Waldst and Kit, have been reported to have healing activity on the *Rattus norvegicus L.* model when administered orally [166]. In the genus Ficus, ten taxa, including the leaves of *Ficus benghalensis L.*, *Ficus microcarpa L.* f., *Ficus hispida L.* f., *Ficus religiosa L.* [167], *Ficus amplissima* Sm. [168], *Ficus exasperata* Vahl [169], *Ficus deltoidea* Jack [170], and *Ficus thonningii* Blume [92] and the stem barks of *Ficus sarmentosa* Buch.-Ham. ex Sm. [171] and *Ficus racemosa L.* [172], have been reported for their healing activity. In the Gardenia genus, the stem barks of *Gardenia gummifera L.* f. [173] and those of *Gardenia ternifolia* Schumach. and Thonn [93] are reported as healing plants. In the Vernonia genus, six taxa with healing properties are reported. These include *Vernonia arborea* Buch.-Ham [syn *Strobocalyx arborea* (Buch.-Ham.) Sch. Bip.] [174]; *Vernonia auriculifera* Hiern [syn *Gymnanthemum auriculiferum* (Hiern) Isawumi] [175]; *Vernonia amygdalina* Delile (syn *Gymnanthemum amygdalinum* (Delile) Sch. Bip.] [176]; *Vernonia conferta* Benth. in Niger Fl. [*Monosis conferta* (Benth.) C. Jeffrey] [177]; *Vernonia leopoldii* Vatke [*Orbivestus leopoldii* (Sch. Bip. ex Walp.) H. Rob.] [178]; and *Vernonia zeylanica* Less [syn *Jeffreycia zeylanica* (L.) H. Rob, S. C. Keeley & Skvarla] [179].

The fact that some taxa in the same genus as the taxa first reported in this study have been reported as healing plants suggests that there is a likelihood that these previously unstudied taxa may also carry healing activity. This review of the literature at the genus level also highlights the taxa *Monotes africanus* and *Polhillides velutina*, for which no taxon in the genus has been reported as a healing plant, and raises the need to determine as soon as possible whether these two taxa would be the first in their genus to have healing properties.

### 4.2. Highlighted Plants

According to their TCIs, *Securidaca longepedunculata* (0.689) for the study as a whole and the category of taxa from households alone; *Allium sativum* (0.086) for taxa from all three sources (households, traditional practitioners, and herbalists); *Acalypha cupricola* (0.081) for taxa from herbalists alone; *Moringa oleifera* (0.043) for taxa derived from conventional practitioners alone; *Sterculia quinqueloba* (0.024) for taxa derived from traditional practitioners and herbalists; and *Acalypha chirindica* (0.047) for taxa derived from conventional practitioners and households are the most consensual taxa in this study, each in its category. Having the highest therapeutic consensus index puts these taxa in a solid and influential position regarding their importance in nonconventional wound care in Lubumbashi. None of these 12 original taxa (class C4) are among the abovementioned plants. This implies that widespread taxa do not necessarily contain a region's originality in healing.

According to their medicinal usual values, *Lantana camara* (13 uses) for the study as a whole and the group of plants originating from traditional practitioners, *Xylopia aethiopica* (10 uses) for taxa originating from TP and herbalists, *Tetradenia riparia* (10 uses) for taxa originating from households, *Euphorbia hirta* (9 uses) for taxa from herbalists, traditional practitioners, and families, *Curcuma longa* (7 uses) for taxa from herbalists, and *Vachellia karroo* (6 uses) for taxa from TP and households, are the plants most used by the population surveyed in the treatment of various diseases. Within this group of 6 taxa, *Lantana camara, Euphorbia hirta, Vachellia karroo,* and *Tetradenia riparia* are the most frequently mentioned in ethnobotanical studies of the region [35, 125, 128, 138, 139], unlike *Xylopia aethiopica* and *Curcuma longa*.

Numerous pathologies are treated by plants inventoried during this study. Fever, malaria, DTIs, diabetes, and STIs, with a RCF ≥50%, are the most frequently cited pathologies (Table S1). However, the five pathologies in descending order of their CMI are diabetes, IGT, malaria, STI, diarrhea, and fever (MCI >0, 11). This ethnobotanical index expresses another facet of the ethnomedicinal characteristics of the region, indirectly translating the specificity of a given region about the therapeutic management of pathologies. As malaria, tuberculosis, lower respiratory tract infections, neonatal disorders, and diarrheal diseases [95] constitute the top 5 most deadly pathologies in the DRC, the results of our study show that the target population has a subsequent phytotherapeutic arsenal to manage two (malaria and diarrhea) of these five pathologies. The population's tendency to resort to nonconventional medicine significantly contributes to the country's healthcare system.

### 4.3. Wound-Healing Recipes

The healing recipes reported in this study are topical. The leaf is the most frequently used organ, and the poultice is the most convenient form of use. These characteristics are not expected in ethnobotanical studies in the region [35, 54, 128], where the leaf is the most widely used organ, decoction is the predominant form, and the oral route is the most common. In a previous study of the country's healing plants [34, 77] and other ethnobotanical reports on wound care [80, 180–184], the leaf was the most commonly used organ, as in the present study. However, the use of leaves is not unequivocal. Similar studies show a preferential use of bark [26, 185, 186].

Similarly, the forms of taxa used with healing potential also vary. In some studies, powder [183, 187], maceration [26], or decoction [77, 188] is predominant, whereas, in our study, poultice is dominant. The latter has the advantage of reducing inflammation, soothing pain, and thoroughly cleansing the skin of toxins and infectious agents. Most of the healing recipes in this study are used externally for wounds; the poultice form and local application are the most suitable.

### 4.4. Characteristics of the Interviewed Population

Unlike previous ethnobotanical studies in Lubumbashi, this study interviewed more women than men [35, 54, 128, 139]. This discrepancy can be explained by the fact that, unlike the present study, the studies above were carried out among practitioners of traditional medicine, predominantly men. In contrast, the present study account felt the general population, which, like the national population in the 40–60 age range, is predominantly female. The fact that most of the subjects were met in households may also justify the preponderance of women. During the household survey, the probability of meeting women was higher than that of meeting men. One of the reasons for this is the responsibility for household chores attributed to women in Congolese society.

## 5. Conclusions

The population of Lubumbashi in Haut-Katanga (DRC), represented both at the household level and by practitioners of traditional medicine and herbalists, uses plants to treat wounds, particularly chronic wounds. Among the taxa used, some are specific to the study region in their use in traditional medicine, particularly in treating wounds. In addition to their role in wound treatment, these taxa are also used to treat a range of common environmental pathologies. These plants offer great potential for the treatment of many other pathologies, such as diabetes. Future studies should focus on validating these medical uses and, where appropriate, identifying the biomolecules responsible for these activities. These taxa are of particular interest and deserve to be domesticated and preserved as part of Miombo's biodiversity.

## Figures and Tables

**Table 1:** Household sample size.

Commune	*N*	*n*_*c*_	*n*_*e*_	Difference (%)
Annexe	749142	384.0	391	1.8
Kamalondo	27911	379.0	389	2.6
Kampemba	526 926	383.9	390	1.6
Katuba	469228	383.8	390	1.6
Kenya	480974	383.9	390	1.6
Lubumbashi	481563	383.9	390	1.6
Rwashi	410 034	383.8	390	1.6
Total	3 145 778	2 682	2 730	1.8

*N*: target population; *n*_*c*_: calculated representative sample; and *n*_*e*_: population surveyed in the field.

**Table 2:** General informations on plants inventoried during the survey and their previous knowledge.

Taxon^Source^ (Family)	NH	Name (ethnicity)	TCI (*n* = 2906)	GT	UP ^MT^	UF^BT^	PRHU	PHA	Medicinal uses (*n* = 70)	MUI (*n* = 70)
*Abelmoschus esculentus* (L.) Moench^z^ (Malvaceae)	KIP000001019	Mulenda (Swahili)	0.042	NEA	Gr^o^	Cataplasm^l^	Turkey: Fr- [37]	*In vivo*-rat-AW: 400 mm^2^-WCR: Fr-12 D [38]	Intestinal worms, dysentery, gastritis, hepatitis, wounds	0.069

*Acalypha chirindica* S.Moore^z^ (Euphorbiaceae)	KIP000001020	Kahenya (Tabwa)	0.047	CA	Rb^n^	Powder^l^	NR	NR	Wounds, jaundice, diabetes	0.042

*Acalypha cupricola* Robyns ex G.A. Levin^z^ (Euphorbiaceae)	KIP000001021	Kabambasheshe (Bemba)	0.081	CA	Lv^o^	Powder^l^	NR	NR	Wound, diabetes	0.028

*Acalypha homblei* De Wild.^z^ (Euphorbiaceae)	KIP000001022	Lwenyi (Luba)	0.074	CA	Lv^q^	Liniment^l^	DRC: Lv [39]	NR	Wound	0.014

*Acalypha ornata* Hochst. ex A. Rich.^z^ (Euphorbiaceae)	KIP000001023	Lusungisungi (Luba-Kat)	0.070	TA	Rb^n^	Ointment^l^	Kenya-Lv- [40]	NR	Wounds, hemorrhoids	0.014

*Acalypha paniculata* Miq.^z^ (Euphorbiaceae)	KIP000001024	Kabobo (Bemba)	0.007	TA	Sb^q^	Powder^l^	NR	NR	Wound, bronchitis	0.028

*Acalypha petiolaris* Hochst^y^ (Euphorbiaceae)	KIP000001432	Kaboko (Bemba)	0.005	TA-SA	R^q^	Liniment^l^	NR	NR	Wound, bronchitis	0.028
					Lv^q^	Powder^l^	NR	NR	Wound, asthma	0.042

*Acalypha psilostachya* Hochst. ex A. Rich.^z^ (Euphorbiaceae)	KIP000001026	Lwenyi (Bemba)	0.006	TA	Sb^q^	Ointment^l^	NR	NR	Wound, pneumonia	0.028

*Acmella caulirhiza* Delile^x^ (Asteraceae)	KIP000001398	Chenda (Shi)	0.007	TA-SA	Lv^o^	Liniment^l^	Mozambique -Sb: [41]	*In vivo*-rat-AW: 625 mm^2^-WCR: 16 D [42]	Wound, gingivitis	0.028

*Aframomum alboviolaceum* (Ridley) K. Schum.^y^ (Zingiberaceae)	KIP000001433	Tondolo (Tabwa)	0.010	TA	R^q^	Cataplasm^l^	NR	NR	Wounds, headaches, hemorrhoids	0.056
					Lv^q^	Ointment^l^	NR	NR	Wound, headache, measles	

*Aframomum angustifolium* (Sonn) K. Schum.^z^ (Zingiberaceae)	KIP000001028	Ntondolo (Mpama)	0.007	TA	PE^q^	Compress^l^	DRC: Lv [34]		Wound, constipation, osteoarthritis	0.042

*Aframomum melegueta* K.^u^ Schum. (Zingiberaceae)	KIP000001341	Masusu (Bemba)	0.070	TA	LV^q^	Compress^l^	Ghana: Lv [43]	*In vivo*-rat-AW: 900 mm^2^-WCR: Se-19 D [44]	Wound, gastritis, diarrhea	0.042

*Agelanthus zizyphifolius* (Engl.) Polhill & Wiens^w^ (Loranthaceae)	KIP000001345	Mbuli (Luba-Kat)	0.042	CA-EA	Rb^m^	Liniment^l^	NR	NR	Wound	0.014

*Ageratum conyzoides* L.^w^ (Asteraceae)	KIP000001346	Kahyole (Shi)	0.015	NEA	Lv^o^	Ointment^l^	Nigeria: Lv and Fw [45]		Wound, burn, osteoarthritis, dyspnea	0.056

*Albertisia villosa* (Exell) Forman^v^ (Menispermaceae)	KIP000001030	Bandegbe (Ngwaka)	0.003	CA	Lv^r^	Powder^l^	NR	NR	Wounds, cataracts, epilepsy	0.042

*Albizia adianthifolia* (Schumach.) W. Wight^v^ (Fabaceae)	KIP000001031	Musase (Luba)	0.004	TA-MA	Lv^m^	Ointment^l^	Cameroun: Sb	NR [46]	Wound, diabetes, syphilis, diarrhea, gonorrhea, and gastrointestinal disorders (GIDs)	0.083

*Albizia antunesiana* Harms^v^ (Fabaceae)	KIP462025883	Musebeya (Shi)	0.015	CA-SA	Sb^n^	Liniment^l^	NR	NR	Wound, diarrhea, gonorrhea	0.042

*Albizia gummifera* (J.F. Gmel.) C.A. Sm.^v^ (Fabaceae)	KIP000001032	Ibange (Rega)	0.001	TA-MA	Lv^m^	Powder^l^	DRC: Lv [34]	NR	Wounds, jaundice, diabetes, headaches, diarrhea	0.069

*Allium sativum* L.^y^ (Amaryllidaceae)	KIP000001430	Ehayi (Nande)	0.087	NEA	Blb^q^	Ointment^l^	Egypt: Blb [47] DRC: Lv [34]	*In vivo*-rat-AW: 300 mm^2^-WCR: Se-16 D [48]	Wounds, hepatitis, hypertension	0.042

*Aloe buettneri* A. Berger^v^ (Asphodelaceae)	KIP000001033	Kizime (Tabwa)	0.001	TA	Lv^q^	Liniment^l^	NR	NR	Burns, vitiligo, malaria	0.042

*Aloe vera* (L.) Burm. f.^u^ (Asphodelaceae)	KIP452120002	Bariba (Tabwa)	0.069	NEA	Lv^q^	Cataplasm^l^	DRC-Lv-: [49] Zimbabwe: Lv [50]	*In vivo*-rat-AW: 100 mm^2^-WCR: Lv >28 D [51]	Diabetes, cancer, burns	0.042

*Anisophyllea boehmii* Engl.^u^ (Anisophylleaceae)	KIP000001025	Lufunga (Tabwa)	0.004	CA	RB	Ointment^l^	NR	NR	Wounds, urinary tract infections, helminthiasis	0.042

*Annona senegalensis* Pers.^v^ (Annonaceae)	KIP452120003	Mulolo (Luba-Kat)	0.017	TA-MA	R, Lv^n^	Cataplasm^l^	DRC: Lv [52] Burkina-Faso: Rb [53]	NR	Wounds, jaundice, diabetes, sickle cell anemia	0.042

*Anonidium mannii* (Oliv.) Engl. & Diels^v^ (Annonaceae)	KIP000001034	Mundenge (Kongo)	0.001	CA	Rb^m^	Powder^l^	NR	NR	Wounds, rheumatism	0.028

*Antidesma membranaceum* Müll. Arg.^v^ (Phyllanthaceae)	KIP000001035	Kifitidi (Kongo)	0.001	TA	Sb^n^	Ointment^l^	NR	NR	Wound, diabetes	0.028

*Antidesma venosum* E. Mey. ex Tul.^v^ (Phyllanthaceae)	KIP293612638	Kifubia (Luba-Kat)	0.001	TA-SA	Lv^m^	Cataplasm^m^	NR		Wound, cough, hypertension, gastritis	0.056

*Baphia capparidifolia* Baker^v^ (Fabaceae)	KIP000001036	Tshikamba (Tshokwe)	0.002	TA-MA	Sb^n^	Cataplasm^m^	Lv, Sb: [54]	NR	Wound, malaria	0.028

*Bidens grantii* Sherff^v^ (Asteraceae)	KIP000001037	Kinukamwilungu (Bemba)	0.003	CA	Lv^o^	Ointment^m^	NR	NR	Wound	0.014

*Bidens pilosa* L.^u^ (Asteraceae)	KIP490924302	Kashisha (Shi)	0.071	NEA	PE^o^	Cataplasm^l^	DRC: Lv [34]	*In vivo*-rat-AW: 706.5 mm^2^-WCR: Lv-17 D [55]	Wound, hypertension, cough, malaria, diabetes, typhoid fever	0.083

*Bobgunnia madagascariensis* (Desv.) J.H. Kirkbr. & Wiersema^y^ (Fabaceae)	KIP002120005	Pampi (Luba-Kat)	0.068	TA	Sb^m^	Cataplasm^l^	NR	NR	Wounds, dysentery, schistosomiasis, malaria	0.069
					Fr^m^	Liniment^l^	NR	NR	Wound, hepatitis	NA

*Brassica oleracea* L.^u^ (Brassicaceae)	KIP000001342	Kabichi (Swahili)	0.071	NEA	Lv^p^	Cataplasm^l^	Romania: Lv [56]	*In vivo*-rat-AW: 300 mm^2^-WCR: Lv-18 D [57]	Wound, diabetes, cancer, obesity, hypertension	0.069

*Bridelia atroviridis* Müll. Arg.^v^ (Phyllanthaceae)	KIP000001038	Kankuku (Luba)	0.008	TA	Rb^m^	Ointment^l^	DRC: Lv [39] Nigeria: Lv [45]	NR	Wound, urethritis, GID, cancer	0.069

*Bridelia duvigneaudii* J. Léonard^v^ (Phyllanthaceae)	KIP000001039	Kalambabwato (Bemba)	0.012	CA	Rb^n^	Powder^l^	DRC: Lv [39]	NR	Wound, diabetes	0.028

*Bridelia ferruginea* Benth.^v^ (Phyllanthaceae)	KIP000001040	Inkuka-i-nsii (Yanzi)	0.011	TA	Sb^m^	Liniment^l^	Nigeria: Rb [58]	*In vivo*-rat-AW: 500 mm^2^-WCR: Sb-17 D [59]	Wound, arthritis, dysentery, constipation, GID	0.069

*Bridelia micrantha* (Hochst.) Baill.^v^ (Phyllanthaceae)	KIP000001041	Mumwenameshi (Bemba)	0.010	TA-SA	Lv^m^	Compress^l^	DRC: Lv [39] RSA: Lv, Rb [60]	*In vivo*-rat-AW: 314 mm^2^-WCR: Lv >15 D [61]	Wound, STI, diarrhea, gingivitis	0.056

*Bridelia scleroneura* Müll. Arg.^v^ (Phyllanthaceae)	KIP000001042	Enjeku (Mbuti)	0.007	TA	Rb^n^	Liniment^m^	NR	NR	Wound, arthritis, GID	0.042

*Cajanus cajan* (L.) Huth^v^ (Fabaceae)	KIP223105500	Ngoliolio (Tabwa)	0.012	NEA	RB^q^	Compress^l^	NR	NR	Wound, GID, gingivitis, malaria	0.056

*Caladium bicolor* (Aiton) Vent.^v^ (Araceae)	KIP000001043	Tokombati (Ngwaka)	0.004	NEA	Tb^q^	Ointment^l^	DRC: Lv [34]	NR	Wound, constipation convulsion, facial paralysis	0.056

*Capsicum frutescens* L.^v^ (Solanaceae)	KIP000001044	Pilipili mbuzi (Swahili)	0.004	NEA	Fr^n^	Liniment^l^	DRC: Fr- [62] Nigeria: Lv [63]	NR	Wound, GID	0.028

*Carduus nyassanus subsp. kikuyorum* (R.E.Fr.) C. Jeffrey^v^ (Asteraceae)	KIP000001045	Mugenbyegembye (Shi)	0.005	CA	Lv^q^	Ointment^m^	Ethiopia: Lv [64]	NR	Wound, GID	0.028

*Carica papaya* L.^u^ (Caricaceae)	KIP041463406	Kipapayi (Swahili)	0.174	NEA	Lx^m^	Liniment^m^	DRC: -R- [34]; Nigeria: Lv [63]	*In vivo*-rat-AW: 78.5 mm^2^-WCR: Lx-20 D [65]	Wound	0.042
					Lv^m^	Ointment^l^		*In vivo*-rat-AW: 314 mm^2^-WCR: Lv-13 D [66]	Wounds, cancer, malaria	NA

*Cassia abbreviata* Oliv.^y^ (Fabaceae)	KIP452120010	Musonkasonka (Bemba)	0.048	TA	R^n^	Ointment^l^	Tanzania: R [67]	NR	Wound, gingivitis, HIV, diabetes	0.056

*Celosia trigyna* L.^y^ (Amaranthaceae)	KIP052120011	Limbila (Lokele)	0.027	TA-SA	R^o^	Liniment^m^	Nigeria: Lv [68]	NR	Wounds, intestinal worms, diarrhea	0.056
					LV	Cataplasm^l^		NR	Wound, mouth ulcer	0,028

*Centella asiatica* (L.) Urb.^u^ (Apiaceae)	KIP000001343	Kurhwirikuguma (Shi)	0.070	TA-SA-MA	LV^q^	Liniment^l^	Egypt: Wp [69]	*In vivo*-rat-AW: 10 mm-WCR: Lv-12 D [70]	Wounds, leprosy, and psoriasis	0.042

*Chamaemelum nobile* (L.) All.^v^ (Asteraceae)	KIP000001047	Mugundun Zimu (Shi)	0.006	NA	Lv^p^	Ointment^l^	Egypt: Fw [71]	*In vivo*-rat-AW: 225 mm^2^-WCR: Fw-5 D [72]	Wound, fever, dysmenorrhea, insomnia, GID, hemorrhoids	0.083

*Chenopodium opulifolium* Schrad ex. W.D.J. Koch & Ziz.^v^ (Chenopodiaceae)	KIP000001048	Gombe Gombe (Shi)	0.014	NA-TA	Lv^o^	Ash^l^	NR	NR	Wound, asthenias, GID	0.042

*Chromolaena odorata* (L.) R.M. King & H. Rob.^v^ (Asteraceae)	KIP000001049	Elengi eye (Lingala)	0.007	NEA	Lv^q^	Ointment^m^	Nigeria: Lv- [73]	*In vivo*-rat-AW: 400 mm^2^-WCR: Lv-15 D [66]	Pl wound, asthenias, GID	0.042

*Cleistanthus polystachyus* Hook. f. ex. Planch^v^ (Phyllanthaceae)	KIP000001050	Mukonde (Bemba)	0.004	CA	Lv, Sb^n^	Powder^l^	NR	NR	Wounds, jaundice, diabetes	0.042

*Coleus esculentus* (N. E. Br) G. Taylor^w^ (Convolvulaceae)	KIP000001347	Matembele (Swahili)	0.009	TA	R^q^	Liniment^l^	NR	NR	Wound, hypertension, diabetes	0.042

*Combretum molle* R. Br. ex G. Don^v^ (Combretaceae)	KIP000001051	Mukonde (Bemba)	0.003	TA	R^n^	Ointment^m^	Mali: [74]	NR	Wound, constipation, headache, gastritis, dysentery, malaria	0.083

*Combretum racemosum* P. Beauv^v^ (Combretaceae)	KIP000001052	Mutsumbi (Yombe)	0.004	TA	Lv^n^	Ointment^m^	DRC: Lv [34]	NR	Wounds, genital urinary infections, helminthes	0.042

*Commelina diffusa* Burm f.^y^ (Commelinaceae)	KIP000001053	Kalambalamba (Luba-Kas)	0.004	TA-SA-MA	Lv^o^	Liniment^m^	Ghana: Lv [75]	NR	Wounds, urinary tract infections, respiratory infections	0.069
					Fw^o^	Compress^m^	NR	NR	Wounds, diarrhea, GID	0.042

*Crassocephalum crepidioides* (Benth.) S. Moore^v^ (Asteraceae)	KIP000001054	Ebolo (Lingala)	0.005	TA-SA-MA	Lv^o^	Cataplasm^m^	Nigeria: Lv [63]	*In vivo*-rat-AW: 144 mm^2^-WCR: Wp 16 D [76]	Wound, tumor, GID	0.042

*Crassocephalum montuosum* (S. Moore) Milne-Redh.^v^ (Asteraceae)	KIP000001055	Cifula (Shi)	0.006	TA-MA	Lv^o^	Ointment^m^	NR	NR	Wound, malaria	0.028

*Crossopteryx febrifuga* (Afzel ex. G. Don) Benth.^v^ (Rubiaceae)	KIP000001056	Mutoshi (Luba-Kas)	0.014	TA	Lv^n^	Liniment^l^	DRC: Lv [77] Benin: [78]	NR	Wound, sickle cell disease, fever, malaria, diarrhea	0.069

*Croton mubango* Müll. Arg.^v^ (Euphorbiaceae)	KIP000001057	Onganga (Tetela)	0.007	CA	Rb^m^	Powder^l^	RSA: Rb [79]	NR	Wound, emaciation, dysentery, diabetes	0.056

*Cucumis melo* L.^w^ (Cucurbitaceae)	KIP000001348	Kikendambaya (Luba-Kat)	0.004	EA	Fr^q^	Liniment^m^	Latin America: Fr, Lx [80]	*In vivo*-rat-AW: 500 mm^2^-WCR: Fr 28 D [81]	Wound, kidney stones, flatulence, leprosy, ascites, anemia, constipation, GID	0.111

*Cucumis sativus* L.^u^ (Cucurbitaceae)	KIP000001344	Liboga (Swahili)	0.106	NEA	Sb^o^	Ointment^m^	NR	NR	Wound, intestinal worms	0.028

*Curcuma longa* L.^v^ (Zingiberaceae)	KIP000001058	Dimputu (Kongo)	0.012	NEA	Se^q^	Ointment^l^	Africa: [27]	*In vivo*-rat-AW: 25 mm^2^-WCR: Fr 18 D [82]	Wound, anorexia, cough, sinusitis, diabetes, hepatitis, rheumatism	0.097

*Cyanthillium cinereum* (L.) H. Rob.^x^ (Asteraceae)	KIP000001399	Kisobo (Bemba)	0.009	TA-MA	F^o^	Ointment^m^	NR	NR	Wounds, rheumatism	0.028

*Dalbergia boehmii* Taub.^v^ (Fabaceae)	KIP452120016	Katembo (Sanga)	0.010	TA	R^n^	Liniment^l^	NR	NR	Wound, malaria, GID, gastric ulcer, cancer	0.069

*Dalbergia nitidula* Welw. ex Baker^v^ (Fabaceae)	KIP000001059	Ndjabilonda (Luba-Kat)	0.009	TA-SA	Rb^n^	Compress^l^	NR	NR	Wound, gingivitis, malaria	0.042

*Dichrostachys cinerea* (L.) Wight & Arn^v^ (Fabaceae)	KIP000001060	Kisanda (Rega)	0.014	TA-SA	Sb^n^	Ointment^l^	RSA: Lv, Sb [83]	NR	Wounds, rheumatism, diabetes, tuberculosis	0.056

*Diospyros mespiliformis* Hochst ex A DC^w^ (Ebenaceae)	KIP000001349	Mugombe (Shi)	0.019	TA	Rb^m^	Ointment^l^	Burkina-Faso: Rb [53]	*In vivo*-rat-AW: 225 mm^2^-WCR: Rb-11 D [84]	Wounds, jaundice, diabetes, intestinal worms	0.056

*Diplorhynchus condylocarpon* (Müll. Arg.) Pichon^w^ (Apocynaceae)	KIP361753009	Mburi (Bemba)	0.027	CA-SA	Sb^n^	Ointment^l^	NR	NR	Wound, GID, fever, snakebite	0.056

*Distephanus biafrae* (Oliv. & Hiern) H. Rob.^x^ (Asteraceae)	KIP000001000	Kande (Tshokwe)	0.008	TA	Rb^n^	Cataplasm^l^	NR	NR	Wound	0.014

*Droogmansia munamensis* De Wild.^w^ (Fabaceae)	KIP000001350	Mununganunga (Bemba)	0.026	CA	R^q^	Cataplasm^l^	NR	NR	Wound, diabetes	0.028

*Dysphania ambrosioides* (L.) Mosyakin & Clemants^v^ (Amaranthaceae)	KIP231226246	Lufianyoka (Bemba)	0.015	NEA	Lv^q^	Cataplasm^l^	DRC: Lv [34]	NR	Wound, malaria, hepatitis, gastritis	0.056

*Ekebergia benguelensis* Welw. ex C. DC.^w^ (Meliaceae)	KIP000001351	Mutuzya (Shi)	0.008	CA	R^m^	Cataplasm^l^	NR	NR	Wound, gastritis, dysentery, epilepsy, STI: sexually transmitted infections	0.069

*Elaeis guineensis* Jacq.^u^ (Aracaceae)	KIP355972071	Mti Ya Ngazi (Swahili)	0.270	TA	R^m^	Cataplasm^l^	West Africa: Lv [85]	*In vivo*-rat-AW: 225 mm^2^-WCR: Wp 20 D [86]	Wound, STI, GID	0.042

*Eleusine indica* (L.) Gaertn^w^ (Poaceae)	KIP000001352	Mutuzya (Shi)	0.007	TA-SA-MA	FW^o^	Cataplasm^l^	Benin: Lv [78]	NR	Wound, HTA, constipation	0.042

*Entada abyssinica* Steud. ex A. Rich^w^ (Fabaceae)	KIP000001353	Cishangishangi (Shi)	0.018	TA-MA	Rb^m^	Liniment^m^	NR	NR	Wound, meningitis	0.028

*Entada africana* Guill. & Perr.^v^ (Fabaceae)	KIP000001061	Lalulalu (Bemba)	0.007	TA	Rb^m^	Ointment^l^	Benin: Lv [78]	NR	Wound, cataract, gastritis, fever, hepatitis	0.069

*Erigeron sumatrensis* Retz.^w^ (Asteraceae)	KIP000001354	Dolodola (Ngwaka)	0.008	NEA	Lv^n^	Powder^l^	NR	NR	Wounds, rheumatism, gout, headaches, dysmenorrhea	0.069

*Eriosema glomeratum* (Guill. & Perr.) Hook. f.^w^ (Fabaceae)	KIP000001355	Zila Wando (Kongo)	0.007	TA	Lv^q^	Ointment^l^	NR	NR	Wound	0.014

*Erythrina abyssinica* Lam. ex DC.^w^ (Fabaceae)	KIP182210666	Kisungwa (Bemba)	0.004	CA-EA	R^m^	Compress^l^	Zimbabwe: Lv, R [87]	*In vivo*-rat-AW: 225 mm^2^-WCR: Wp 16 D [86]	Wound, HIV, diabetes, malaria, GID, diarrhea	0.083

*Euphorbia heterophylla* L.^w^ (Euphorbiaceae)	KIP000001356	Butonvi (Bemba)	0.004	NEA	Lv^o^	Cataplasm^l^	DRC: [39] Nigeria: Lv [88]	*In vivo*-rat-AW: 49 mm^2^-WCR: Wp 24 D [88]	Wound, constipation, GID	0.042

*Euphorbia hirta* L.^y^ (Euphorbiaceae)	KIP501400542	Butonvitonvi (Bemba)	0.060	NEA	PE^o^	Ointment^l^	Nigeria: Lv [89]	*In vivo*-rat-AW: 490 mm^2^-WCR: Lv 16 D[90]	Wound, cough, bronchitis, asthma, amoeba, intestinal worms, GID, gastritis, dysentery, jaundice, STI, tumors, pneumonia	0.125

*Euphorbia hypericifolia* L.^w^ (Euphorbiaceae)	KIP000001357	Hivumba (Hemba)	0.014	NEA	Wp^o^	Powder^l^	DRC: [39]	NR	Wound, STI, pneumonia, bronchitis	0.056

*Euphorbia inaequilatera* Sond.^w^ (Euphorbiaceae)	KIP000001358	Kalong (Rund)	0.001	NA-TA-SA	Lv^o^	Powder^l^	DRC: [39]	NR	Wound, eye infections, STI	0.042

*Euphorbia terracina* L.^w^ (Euphorbiaceae)	KIP000001359	Umafa (Bemba)	0.003	NA	Lv^q^	Cataplasm^l^	NR	NR	Wound, respiratory infections, STI	0.042

*Euphorbia tirucalli* L.^w^ (Euphorbiaceae)	KIP000001360	Karhoza (Shi)	0.004	TA-SA-MA	Lv^o^	Ointment^l^	DRC: [39]	NR	Wounds, hemorrhoids, STI	0.042

*Ficus ampelos* Burm. f.^w^ (Moraceae)	KIP000001361	Hiakasi (Kongo)	0.005	NEA	Sv^m^	Powder^l^	NR	NR	Wound, GID	0.028

*Ficus sur* Forssk.^y^ (Moraceae)	KIP539378804	Kikuyu (Luba)	0.063	TA-SA	Wp^m^	Cataplasm^l^	Nigeria: Lv [91]	*In vivo*-rat-AW: 200 mm^2^-WCR: Lv 18 D [91]	Wound, peptic ulcer, diabetes, GID, STI, tuberculosis	0.083

*Ficus thonningii* Blume^w^ (Moraceae)	KIP000001362	Musambi (Bemba)	0.004	TA-SA	RB^m^	Cataplasm^m^	Benin Lv, Rb: [78]	*In vivo*-rat-AW: 500 mm^2^-WCR: Lv 14 D [92]	Wound, dysentery, constipation	0.042

*Flueggea virosa* (Roxb. ex. Willd.) Royle^w^ (Phyllanthaceae)	KIP000001363	Musangala (Luba-Kat)	0.006	TA-SA-MA	Lv, Rb^n^	Cataplasm^m^	Benin: Lv, Rb: [78]	NR	Wound, snakebite, STI, rheumatism, sexual dysfunction	0.069

*Garcinia huillensis* Welw. ex Oliv.^w^ (Clusiaceae)	KIP000001364	Mungindu (Tshokwe)	0.007	CA-EA	Lx^n^	Ointment^l^	NR	NR	Wound, angina pectoris, jaundice, diabetes, STI, measles, bronchitis, pneumonia	0.111

*Gardenia imperialis* K. Schum.^w^ (Rubiaceae)	KIP000001365	Munamba (Bemba)	0.008	TA	Sb^m^	Liniment^m^	NR	NR	Wound, anxiety	0.028

*Gardenia ternifolia* Schumach. & Thonn.^w^ (Rubiaceae)	KIP000001366	Kidia (Kongo)	0.009	TA	Sb^n^	Powder^l^	Benin: Lv, Rb [78]	*In vivo*-rat-AW: 250 mm^2^-WCR: Sb-18 D [93]	Wounds, malaria, hypertension, diabetes, coughs, asthma, rheumatism, diarrhea, tooth decay, leprosy, hepatitis, hemorrhoids, and cancer	0.181

*Gymnanthemum amygdalinum* (Delile) Sch. Bip.^x^ (Asteraceae)	KIP312286606	Kilolokonjo (Swahili)	0.004	TA	Lv^n^	Compress^l^	DRC: Lv [34] Nigeria: [45]	NR	Wound, fever, cough, constipation, TSI	0.069

*Harungana madagascariensis* Lam. ex Poir.^w^ (Hypericaceae)	KIP139657408	Mukuta (Tabwa)	0.009	TA-MA	Sb^n^	Cataplasm^l^	Nigeria: [94]	*In vivo*-rat-AW:490 mm^2^-WCR: Rb-NR [94]	Wound, anemia, malaria, GID	0.056

*Heinsia crinita* (Wennberg) G. Taylor^w^ (Rubiaceae)	KIP000001367	Kinkete (Kongo)	0.010	TA	Lv^n^	Ointment^m^	NR	NR	Wound, sexual dysfunction, fever, malaria	0.056
					R^n^	Ointment^l^	NR	NR	Wound, fever, malaria	0.042

*Hibiscus surattensis* L.^w^ (Malvaceae)		Dongodongo (Lingala)	0.004	TA	Lv^o^	Ointment^m^	NR	NR	Wound, urethritis, STI	0.042

*Hymenocardia acida* Tul.^w^ (Phyllanthaceae)	KIP464414491	Kapempe (Bemba)	0.038	TA	R^n^	Ointment^l^	DRC: Rb [34]	NR	Wound, diarrhea, dysentery, GID	0.056

*Hypoestes triflora* (Forssk.) Roem. & Schult.^w^ (Acanthaceae)	KIP000001368	Mageru (Shi)	0.014	TA	Lv^q^	Ointment^l^	NR	NR	Wound, anemia, HIV	0.042

*Imperata cylindrica* (L.) Raeusch.^w^ (Poaceae)	KIP000001369	Lubamba (Swahili)	0.017	NA-TA-SA-MA	Wp^q^	Liniment^l^	DRC: Lv [34]	NR	Wound, peptic ulcer, diabetes, HIV, hepatitis	0.069

*Jacobaea maritima* (L.) Pelser & Meijden^x^ (Asteraceae)	KIP000001001	Esenesha (Shi)	0.005	NA	R^q^	Liniment^l^	NR	NR	Wound, fever	0.028

*Jatropha curcas* L.^u^ (Euphorbiaceae)	KIP000001437	Ntondondimba (Bemba)	0.314	NEA	L^n^	Ointment^l^	DRC: Lv [95] Nigeria: Lv [63]	*In vivo*-rat-AW: 177 mm^2^-WCR: Lv-18D [96]	Wound, fever, diabetes	0.014

*Julbernardia paniculata* (Benth.) Troupin^w^ (Fabaceae)	KIP452120019	Cigebu (Shi)	0.018	CA	RB^m^	Liniment^m^	Angola: Lv, Rb [97]	*In vitro* (NHDF)-AW: NA-WCR Rb, Lv-NA [97]	Wound, malaria	0.028

*Justicia insularis* T. Anderson^w^ (Acanthaceae)	KIP000001370	Luhe (Luba-Kat)	0.027	WE-CA	SB^o^	Cataplasm^l^	NR	NR	Wound, malaria, diabetes	0.042

*Kalaharia uncinata* (Schinz) Moldenke^v^ (Lamiaceae)	KIP000001062	Ntagalala (Bemba)	0.008	CA-SA	Lv^n^	Cataplasm^m^	NR	NR	Wound, malaria	0.028

*Khaya anthotheca* (Welw.) C. DC.^w^ (Meliacea)	KIP000001371	Kasembe Sembe (Luba-Kat)	0.008	TA	Lv^m^	Cataplasm^m^	NR	NR	Wound, pneumonia, GID, sexual dysfunction	0.056

*Khaya nyasica* Stapf ex Baker f.^w^ (Meliaceae)	KIP000001372	Mbamba (Luba-Kat)	0.015	EA	Sb^m^	Cataplasm^l^	DRC: Sb- [54]	NR	Wounds, jaundice, diabetes	0.042

*Kigelia africana* (Lam.) Benth.^w^ (Bignoniaceae)	KIP227674231	Kivungwila (Tabwa)	0.021	TA	R^m^	Cataplasm^l^	Ghana: Lv, Rb [98]	*In vivo*-rat-AW: 1256 mm^2^-WCR: Rb 19 D [98]	Wound, impetigo	0.028

*Landolphia congolensis* (Stapf) Pichon^w^ (Apocynaceae)	KIP000001373	Musange (Rega)	0.027	CA	Rb^q^	Cataplasm^l^	NR	NR	Wound	0.014

*Landolphia kirkii* Dyer ex Hook. f.^w^ (Apocynaceae)	KIP000001027	Mabungo (Bemba)	0.046	CA-EA-SA	Rb^n^	Liniment^m^	NR	NR	Wound, malaria, diarrhea	0.042

*Lantana camara* L.^w^ (Verbenaceae)	KIP452120021	Kushukashuha (Shi)	0.004	NEA	Se^n^	Ointment^l^	NR	*In vivo*-rat-AW: 300 mm^2^-Rb-WCR: NR D [99]	Wounds, malaria, cancer, chickenpox, measles, asthma, ulcers, eczema, tumors, hypertension, fevers, cataracts, dysmenorrhea, rheumatism	0.194

*Luffa aegyptiaca* Mill.^w^ (Cucurbitaceae)	KIP247475402	Cyangwe (Shi)	0.005	NEA	Fr^q^	Ointment^l^	NR	NR	Wound, sinusitis	0.028

*Manihot esculenta* Crantz^u^ (Euphorbiaceae)	KIP000005804	Lulundu (Luba-Kat)	0.353	NEA	Lv^n^	Cataplasm^m^	DRC: R [39]	*In vivo*-rat-AW: 400 mm^2^-WCR: LV >21 D [100]	Wound, hypertension, headaches	0.042

*Maprounea africana* Müll. Arg.^w^ (Euphorbiaceae)	KIP000001374	Kafulumume (Hemba)	0.001	TA	Lv, Fr^n^	Cataplasm^l^	DRC: Sev [39]	NR	Wound, headache, GID, cough	0.056

*Markhamia lutea* (Benth.) K. Schum.^w^ (Bignoniaceae)	KIP000001375	Musabo (Shi)	0.002	TA	Rb^m^	Powder^m^	NR	NR	Wound, headache, convulsion, cough	0.056

*Memecylon flavovirens* Baker^w^ (Melastomataceae)	KIP000001376	Mifisha (Bemba)	0.012	CA	Rb, Sb^n^	Liniment^l^	DRC: Fr [54]	NR	Wound, herpes	0.028

*Mitragyna stipulosa* (DC.) Kuntze^w^ (Rubiaceae)	KIP000001377	Mumpa (Bemba)	0.004	TA	Lv, Sb^m^	Cataplasm^l^	NR	NR	Wound	0.014

*Monotes africanus* A. DC.^w^ (Dipterocarpaceae)	KIP000001378	Kipampa (Bemba)	0.003	TA	Sb^m^	Cataplasm^l^	NR	NR	Wound, HIV, fever	0.042

*Monotes katangensis* (De Wild) De Wild.^w^ (Dipterocarpaceae)	KIP452120022	Kimpampa (Bemba)	0.034	CA-EA	Rb^m^	Cataplasm^l^	NR	NR	Wound, typhoid fever, diarrhea	0.042

*Moringa oleifera* Lam^w^ (Fabaceae)	KIP318101663	Amba (Luba-Kat)	0.043	NEA	Lv^m^	Liniment^l^	DRC: Lv [34]	*In vivo*-rat-AW: 30 mm^2^-WCR: Lv-12 D [101]	Wound, sexual dysfunction	0.028

*Mucuna poggei* Taub.^w^ (Fabaceae)	KIP000001379	Kudikudi (Bemba)	0.004	TA	Lv^r^	Liniment^m^	NR	NR	Wound, schistosomiasis, STI	0.042

*Musa* × *paradisiaca* L.^u^ (Musaceae)	KIP000000342	Mugomba (Swahili)	0.642	NEA	Lv, T^q^	Ointment^l^	DRC: Lv [77]	*In vivo*-rat-AW: 12.56 mm^2^-UP: Lv WCR: 16 D [102]	Wounds, dysentery, bronchitis, diarrhea	0.056

*Nauclea latifolia* Sm.^w^ (Rubiaceae)	KIP000001380	Kienga (Kongo)	0.004	TA	Lv^n^	Powder^l^	NR	*In vivo*-rat-AW: 300 mm^2^-WCR: Rb 24 D [103] Lv 21 D [100]	Wounds, fever, tooth decay, malaria, dysentery, diarrhea, and epilepsy	0.097

*Nicandra physalodes* (L.) Gaertn.^w^ (Solanaceae)	KIP000001381	Songua (Lamba)	0.004	NEA	Lv^o^	Cataplasm^l^	NR	NR	Wound, helminth, fever	0.042

*Ochna schweinfurthiana* F. Hoffm.^w^ (Ochnaceae)	KIP000001382	Jihoni (Bemba)	0.005	TA	R^m^	Liniment^l^	Benin: Rb [78]	NR	Wound, malaria	0.028

*Ocimum gratissimum* L.^w^ (Lamiaceae)	KIP000001383	Lwenyi (Bemba)	0.006	TA-MA	L^q^	Powder^l^	Ghana: Lv [43]	*In vivo*-rat-AW: 120 mm^2^-WCR: Lv 16 D [104]	Wound, diarrhea, anemia, diabetes, cancer	0.069

*Oldfieldia dactylophylla* (Welw. ex Oliv.) J Léonard^w^ (Picrodendraceae)	KIP000001384	Muonga (Swahili)	0.006	CA-EA	Sb^m^	Cataplasm^l^	NR	NR	Wounds, jaundice, diabetes	0.042

*Parinari curatellifolia* Planch. ex Benth.^w^ (Chrysobalanaceae)	KIP000001385	Mpundu (Bemba)	0.012	TA-MA	Sb^m^	Cataplasm^l^	Togo: Lv [105]	NR	Wound, cancer, pneumonia, fever	0.056

*Paullinia pinnata* L.^w^ (Sapindaceae)	KIP000001386	Tongbisisa (Ngbandi)	0.023	TA-MA	Lv, Rb^n^	Ointment^l^	Benin: Lv, Rb [78]	*In vivo*-rat-AW: 225 mm^2^-WCR-AP 21 D [106]	Wound, arthritis	0.028

*Penianthus longifolius* Miers^w^ (Menispermaceae)	KIP000001387	Songbolo (Ngwaka)	0.007	CA	Rb^n^	Ointment^l^	DRC: Rb [34]	NR	Wounds, jaundice, diabetes	0.042

*Phyllanthus muellerianus* (Kuntze) Exell^y^ (Phyllanthaceae)	KIP277202454	Mulembalemba (Hemba)	0.031	TA	Lv, Rb, Fw^m^	Ointment^l^	DRC: Lv, R Fr [39]	*In vivo*-rat-AW: 400 mm^2^ WCR-AP 14 D [107]	Wound, constipation, dysentery	0.042

*Phyllanthus niruri* L.^w^ (Phyllanthaceae)	KIP000001388	Kapondo (Songe)	0.004	NEA	R^q^	Ointment^l^	DRC: Lv [34]	*In vivo*-rat-AW: 400 mm^2^ WCR-AP 12 D[108]	Wound, constipation, dysentery	0.056

*Phyllanthus ovalifolius* Forssk.^w^ (Phyllanthaceae)	KIP000001389	Luheyamafumu (Luba-Kas)	0.003	TA	PE, RB^n^	Cataplasm^l^	NR	NR	Peptic ulcer, wound, peptic ulcer, wound	0.042

*Phyllanthus parvulus* Sond.^w^ (Phyllanthaceae)	KIP000001390	Mushindanga (Bemba)	0.004	CA-SA	R, LV^q^	Cataplasm^l^	NR	NR	Wound, HTA	0.028

*Piliostigma thonningii* (Schum.) Milne-Redh^w^ (Fabaceae)	KIP452120029	Kifumbe (Bemba)	0.004	TA	RB^n^	Powder^l^	Benin: LV, Rb [78]	*In vivo*-rat-AW: 225 mm^2^ WCR-AP 15 D [109]	Wound, arthritis, malaria, diarrhea, gingivitis	0.069

*Polhillides velutina* (Willd.) H. Ohashi & K. Ohashi^v^ (Fabaceae)	KIP000001063	Irhuza (Shi)	0.013	TA	R^o^	Cataplasm^l^	NR	NR	Wound	0.014

*Pseudolachnostylis maprouneifolia* Pax^w^ (Fabaceae)	KIP000001391	Musalya (Bemba)	0.005	TA	RB^n^	Cataplasm^l^	DRC: Sb, Rb [39] Mozambique: Rb [110]	NR	Wound, cancer, schistosomiasis	0.042

*Psidium guajava* L.^w^ (Myrtaceae)	KIP000301861	Mapera (Swahili)	0.006	NEA	LV^m^	Cataplasm^m^	Nigeria: Lv [111]	*In vivo*-mice-AW: 7.065 mm^2^ WCR-AP 14 D [112]	Wound, diarrhea, dysentery, GID	0.056

*Psorospermum corymbiferum* Spach.^w^ (Hypericaceae)	KIP000001392	Mukuta (Luba-Kat)	0.006	WE-CA	Lv^n^	Powder^m^	NR	NR	Wound, malaria	0.028

*Psorospermum febrifugum* Spach.^w^ (Hypericaceae)	KIP510193316	Mulemba (Luba-Kat)	0.004	TA	Rb^n^	Cataplasm^m^	Zimbabwe: Rb [50]	NR	Wound, fever, diarrhea	0.042

*Pterocarpus angolensis* DC.^w^ (Fabaceae)	KIP000001393	Mulombwa (Bemba)	0.005	TA	RB^n^	Powder^m^	DRC: Rb [113] Angola: Lv, Rb [97]	*Ex vivo* NHDF-AW: NA-WCR-NA-UP: Rb, Lv [97]	Wound, sexual dysfunction, fever	0.042

*Ricinus communis* L.^w^ (Euphorbiaceae)	KIP396088433	Lundondo (Hemba)	0.006	EA	Lv^m^	Ointment^l^	DRC: Lv [39] Nigeria: Se [45]	*In vitro*-MDCK cell-AW: NA-WCR: Lv-NA [114]	Wound, insomnia, rheumatism	0.042

*Salvia officinalis* L.^w^ (Lamiaceae)	KIP000001394	Salmiya (Bemba)	0.012	NEA	Lv^q^	Cataplasm^l^	NR	*In vivo*-mice-AW: 314 mm^2^ WCR-15 D [115]	Wound, rheumatism, gout, diabetes, tumor, diarrhea	0.083

*Securidaca longepedunculata* Fresen^u^ (Polygonaceae)	KIP000001438	Muyeye (Bemba)	0.689	TA-SA	RB^n^	Cataplasm^m^	DRC: Lv [34]	*In vivo*-mice-AW: 400 mm^2^ WCR-21D [116]	Wound, headache, constipation	0.042

*Senna occidentalis* (L.) Link^u^ (Fabaceae)	KIP231974463	Mushingemanjoka (Mashi)	0.192	NEA	R^q^	Powder^l^	Tanzania: Lv, R [117]	*In vivo*-mice-AW: 1962.5 mm^2^ WCR-17D [118]	Wound, constipation, diabetes, malaria, GID	0.069

*Senna petersiana* (Bolle) Lock^x^ (Fabaceae)	KIP000001002	Kundekunde (Fulero)	0.004	TA-SA-MA	R^n^	Powder^l^	NR	NR	Wound, diabetes, GID	0.042

*Senna siamea.* (Lam.) H. S. Irwin & Barneby^x^ (Fabaceae)	KIP000001003	Mutarabanyi (Shi)	0.004	NEA	R^m^	Ointment^m^	NR	NR	Wound, GID, jaundice, fever, typhoid fever	0.069

*Senna singueana* (Delile) Lock^x^ (Fabaceae)	KIP000001004	Munungalino (Sanga)	0.007	TA-SA	R^n^	Ointment^l^	NR	NR	Wounds, jaundice, diabetes, rheumatism, STI	0.069

*Shirakiopsis elliptica* (Hochst.) Esser^w^ (Euphorbiaceae)	KIP000001396	Loniangu (Kongo) Mukondokondo (Bemba)	0.001	TA-SA	Lv, RB^m^	Liniment^l^	NR	NR	Wounds, jaundice, diabetes, ascites	0.056
					Lv^m^	Ointment^m^	DRC: Lv [39]	NR	Wound, ascites, constipation	0.042

*Smilax anceps* Willd.^x^ (Smilacaceae)	KIP000001005	Kikalala, baka (Kongo)	0.011	TA-SA-MA	Lv, Rz^q^	Cataplasm^m,l^	NR	NR	Wounds, arthritis, rheumatism	0.042

*Steganotaenia araliacea* Hochst.^x^ (Apiaceae)	KIP000001006	Kulandosi (Kongo)	0.009	TA-SA	RB^n^	Cataplasm^l^	NR	NR	Wound, constipation, diabetes	0.042

*Stephania abyssinica* (Quart.-Dill. & A. Rich.) Walp^x^ (Menispermaceae)	KIP000001007	Tumbatumba (Hemba)	0.012	TA-SA	R^n^	Cataplasm^l^	Ethiopia: R [119]	*In vivo*-mice-AW: 300 mm^2^ WCR: R-16D [119]	Wound, hypertension, diabetes	0.042

*Sterculia quinqueloba* (Garcke) K. Schum.^x^ (Sterculiaceae)	KIP000001008	Mwabi (Luba-Kat)	0.024	CA	B^m^	Ointment^l^	NR	NR	Wound, gastritis	0.028

*Syzygium guineense* (Willd.) DC.^x^ (Myrtaceae)	KIP000001009	Mugote (Swahili)	0.007	TA-SA	SB^m^	Powder^l^	Mali: Lv [120]	NR	Wound, cough, asthma	0.042

*Terminalia mollis* M.A. Lawson^x^ (Chrysobalanaceae)	KIP452120033	Kibobo (Bemba)	0.013	TA	Lv^m^	Cataplasm^l^	NR	NR	Wound, diarrhea, dysentery, GID	0.056

*Tetradenia riparia* (Hochst.) Codd.^u^ (Lamiaceae)	KIP370001297	Mutuzo (Bemba)	0.306	TA-SA	Lv^n^	Compress^l^	RSA: Lv [121]	*In vitro*-APPA-AW: NA; WCR: Lv-%7 [121]	Wound, respiratory infections, headache, malaria, gastritis, cough, fever, angina, diarrhea, gingivitis	0.139

*Triumfetta rhomboidea* Jacq.^x^ (Malvaceae)	KIP000001010	Mulangalanga (Swahili)	0.019	TA-SA	Rb^q^	Ointment^l^	NR	NR	Diarrhea, gastrointestinal ulcer, tumor	0.042

*Uapaca acuminata* (Hutch.) Pax & K. Hoffm.^x^ (Phyllanthaceae)	KIP000001011	Musela (Rega)	0.008	CA	Sb^m^	Ointment^l^	NR	NR	Wound, tumor	0.028

*Uapaca kirkiana* Müll. Arg.^u^ (Phyllanthaceae)	KIP000001439	Masuku (Bemba)	0.234	CA	Sb^n^	Ointment^l^	DRC: Rb [39]	NR	Wound, diabetes, GID, dysentery	0.056

*Uapaca nitida* Müll. Arg.^y^ (Phyllanthaceae)	KIP000001431	Musonkolobe (Bemba)	0.010	TA	Rb^n^	Ointment^l^	DRC: Rb [39]	NR	Wound, STI, GID	0.042

*Uapaca pilosa* Hutch.^x^ (Phyllanthaceae)	KIP000001012	Mupangwa (Bemba)	0.005	TA	Rb^n^	Liniment^l^	DRC: Rb [39]	NR	Wound, dysentery, constipation, diabetes	0.056

*Uapaca robynsii* De Wild.^x^ (Phyllanthaceae)	KIP000001013	Sambi (Bemba)	0.002	CA	Rb^n^	Cataplasm^l^		NR	Wound, dysentery, constipation, diabetes	0.056

*Uapaca sansibarica* Pax^x^ (Phyllanthaceae)		Mutankola (Bemba)	0.018	TA	Rb^m^	Cataplasm^l^	DRC: Rb [39]	NR	Wound, diarrhea	0.028

*Vachellia karroo* (Hayne) Banfi & Galasso^z^ (Fabaceae)	KIP000001029	Munganushi (Bemba)	0.046	SA	Sb^m^	Ointment^l^	RSA: Lv [122]	NR	Wound, sexual dysfunction, diarrhea, dysentery, diabetes	0.083

*Vernonia excelsa* Jongkind^x^ (Asteraceae)	KIP000001014	Ruhombo (Shi)	0.003	CA	Lv^r^	Cataplasm^l^	NR	NR	Wound, diabetes	0.028

*Xylopia aethiopica* (Dunal) A. Rich.^x^ (Annonaceae)	KIP000001015	Nsombo (Kongo)	0.004	TA	Fr^m^	Ointment^l^	Benin: Se [78]	NR	Wounds, coughs, hemorrhoids, uterine fibroids, malaria, syphilis, amenorrhea, diabetes and dysentery, sickle cell anemia	0.139

*Zanha africana* (Radlk.) Exell^w^ (Sapindaceae)	KIP000001397	Kalayi (Tabwa)	0.001	CA	Rb^n^	Ointment^m^	NR	NR	Wound, headache, pneumonia, fever, rheumatism	0.069

*Zanthoxylum chalybeum* Engl.^x^ (Rubiaceae)	KIP000001016	Pupwe (Bemba)	0.003	CA-EA	R^n^	Powder^l^	DRC: R [54]	NR	Wound, malaria, sickle cell disease, cough	0.056

*Ziziphus abyssinica* Hochst ex A. Rich.^x^ (Rhamnaceae)	KIP000001017	Mukobakoba (Luba-Kas)	0.022	TA	R^n^	Cataplasm^l^		*In vivo*-mice-AW: 300 mm^2^ WCR: R-13D [123]	Wound, fever, tuberculosis	0.042

*Ziziphus mucronata* Willd.^x^ (Rhamnaceae)	KIP000001018	Nkakona (Bemba)	0.004	TA-SA-MA	Lv, Rb^n^	Cataplasm^l^	Mozambique: Lv [124] Zimbabwe: Lv, R [50]	NR	Wound, gingivitis, tuberculosis, STI, sexual dysfunction	0.069

NH: Herbarium number; TCI: therapeutic consensus index; GT: geographical type; UP: used part; MT: morphological type; FU: form of use; PRHU: previously reported healing use; PHA: proven healing activity: experimental model, wound surface, wound contraction rate of the part used; IUM: medicinal use index; AW: area of the wound; WCR: wound contraction rate; STI: sexually transmitted infection; GID: gastrointestinal disorders; traditional healers and herbalists (u); traditional healers (v); households (w); herbalists (x); traditional healers and herbalist & households (y); traditional healers and households (z); tree (m); shrub (n); annual herb (o); biennial herb (p); perennial herb (q); liana (r); k: acute wound; l: chronic wound; Lv: leaves; R: roots; Rb: root bark; Sb: stem bark; St: stem; AP: aerial parts; Se: seeds; Fw: flowers; Nx: nuts; Fr: fruit; Lx: latex; Blb: bulb; Wp: whole plant; NHDF: normal human dermal fibroblasts; APPA: assay/protein precipitating activity. The pharmacological studies reported on the taxa in this study are in vivo ethnopharmacological studies in which the extract is applied locally to the wound as a 10% ointment. Where no in vivo study is reported, but there is another study carried out on the taxon concerned, this is also reported, giving the experimental model used.

**Table 3:** Characteristics of herbal healing recipes used in blends.

No.	Taxa 1	Taxa 2	UP (ratio)	Formulation	TCI (*n* = 2906)
1	*Acalypha homblei*	*Euphorbia hirta*	Lv-Lv (1 ÷ 2)	Ointment	0.0282
2	*Albizia antunesiana*	*Baphia capparidifolia*	R-R (1 ÷ 1)	Cataplasm	0.0193
3	*Cassia abbreviata*	*Phyllanthus muellerianus*	R-Rb (1 ÷ 2)	Liniment	0.0083
4	*Entada abyssinica*	*Ageratum conyzoides*	Lv-R (2 ÷ 1)	Cataplasm	0.0124
5	*Euphorbia hirta*	*Ageratum conyzoides*	Lv-Lv (2 ÷ 1)	Cataplasm	0.0447
6	*Flueggea virosa*	*Garcinia huillensis*	Rb-Rb (2 ÷ 1)	Cataplasm	0.0083
7	*Gymnanthemum amygdalinum*	*Senna singueana*	Lv-R (1 ÷ 1)	Ointment	0.0041
8	*Khaya nyasica*	*Ekebergia benguelensis*	R-Sb (1 ÷ 1)	Liniment	0.0224
9	*Moringa oleifera*	*Nauclea latifolia*	Lv-Lv (2 ÷ 1)	Cataplasm	0.3248
10	*Moringa oleifera*	*Garcinia huillensis*	Lv-Rb (1 ÷ 1)	Ointment	0.3365
11	*Paullinia pinnata*	*Heinsia crinita*	Rb-R (2 ÷ 1)	Ointment	0.0034

MCI: medicinal consensus index; UP: part used; Rb: root bark; Lv: leaves; R: roots; Sb: stem bark.

**Table 4:** Sociodemographic characteristics of people consulted.

Class	Category	Household (*n* = 2730)		Herbalists (*n* = 48)		Traditional healers (*n* = 128)		Total% (*n* = 2906)
		*E*	%	*E*	%	*E*	%	
Age	20–30	68	2.5	2	4.2	7	5.5	2.6
	30–40	458	16.8	3	6.3	19	14.8	16.5
	40–50	215	7.9	8	16.7	29	22.7	8.7
	50–60	1230	45.1	30	62.5	64	50.0	45.6
	>60	759	27.8	5	10.4	9	7.0	26.6

Experience (year range)	0–5	218	8.0	8	16.7	11	8.6	8.2
	6–10	328	12.0	4	8.3	62	48.4	13.6
	11–15	410	15.0	24	50.0	28	21.9	15.9
	16–20	1638	60.0	5	10.4	21	16.4	57.3
	21–25	136	5.0	7	14.6	6	4.7	5.1

Profession	Retailer	50	1.8	0	0.0	25	19.5	2.6
	Civil servant	993	36.4	0	0.0	0	0.0	34.2
	Liberal	157	5.8	0	0.0	3	2.3	5.5
	Housekeeper	1530	56.0	0	0.0	0	0.0	52.6
	Traditional practitioner	0	0.0	0	0.0	100	78.1	3.4
	Herbalist	0	0.0	48	100.0	0	0.0	1.7

Type	Female	1557	57.0	39	81.3	44	34.4	56.4
	Male	1173	43.0	9	18.8	84	65.6	43.6

Residence	Annex	391	14.3	16	33.3	32	25.0	15.1
	Kamalondo	389	14.2	4	8.3	14	10.9	14.0
	Kampemba	390	14.3	4	8.3	10	7.8	13.9
	Katuba	390	14.3	4	8.3	26	20.3	14.5
	Kenya	390	14.3	6	12.5	14	10.9	14.1
	Lubumbashi	390	14.3	8	16.7	15	11.7	14.2
	Rwashi	390	14.3	6	12.5	17	13.3	14.2

Level of education	None	437	16.0	10	20.8	24	18.8	16.2

Class	Primary	951	34.8	6	12.5	20	15.6	33.6
	Vocational	167	6.1	10	20.8	12	9.4	6.5

Age	Secondary	1047	38.4	21	43.8	66	51.6	39.0
	University	128	4.7	1	2.1	6	4.7	4.6

By experience, we mean the number of years the target population has been using medicinal plants to treat their illnesses.

## Data Availability

Some of the data underpinning the results of this study are available in this article and some in the supplementary material.

## Supplementary Materials

Supplementary Materials Table S1: other therapeutic medications from inventoried plants. Table S1 provides additional data on the different pathologies treated by the plants inventoried in our study, the number of plants used for this purpose, the resulting medicinal citation index, the number of respondents who cited the pathology, and the different taxa involved in the treatment of this pathology.
