# Supplementary material for: Ethnomedical Knowledge of Plants Used in Nonconventional Medicine for Wound Healing in Lubumbashi, Haut-Katanga Province, DR Congo
Source: ScientificWorldJournal. 2024 Aug 12;2024:4049263. doi: 10.1155/2024/4049263 (PMC11458279; doi:10.1155/2024/4049263)
Supplement: Supplementary Materials — Table S1: other therapeutic medications from inventoried plants. Table S1 provides additional data on the different pathologies treated by the plants inventoried in our study, the number of plants used for this purpose, the resulting medicinal citation index, the number of respondents who cited the pathology, and the different taxa involved in the treatment of this pathology. [file 4049263.f1.docx]

# Supporting Information (Supplementary data)

**Manuscript number**: ID 4049263

**Title**: Ethnomedical knowledge of plants used in non-conventional medicine for wound healing in Lubumbashi, Haut-Katanga Province, DR. Congo

**Article type** : Research Paper

**Corresponding Author** : Prof Bashige Chiribagula Valentin, [bashige.chiribagula@unilu.ac.cd](mailto:bashige.chiribagula@unilu.ac.cd)

Table S1 provides additional data on the different pathologies treated by the plants inventoried in our study, the number of plants used for this purpose, the resulting medicinal citation index, the number of respondents who cited the pathology, and the different taxa involved in the treatment of this pathology.

**Table S1**: Other therapeutic medications from inventoried plants.

| **Medical uses (nu=70)** | **n_t_** | **MCI (n=166)** | **N_c_** | **RCF (N=2906)** | **Plant species** | |
| --- | --- | --- | --- | --- | --- | --- |
| Diabetes mellitus  | 43 | 0.259 | 1721 | 59.22 | *Curcuma longa, Gardenia ternifolia, Xylopia aethiopica, Diospyros mespiliformis, Albizia adianthifolia , Uapaca kirkiana , Uapaca pilosa, Penianthus longifolius, Annona senegalensis, Jatropha curcas, Bridelia duvigneaudii, Bidens pilosa, Phyllanthus niruri, Albizia gummifera, Imperata cylindrica, Stephania abyssinica, Ocimum gratissimum, Ficus sur, Acalypha chirindica, Acalypha cupricola, Antidesma membranaceum, Cleistanthus polystachyus, Coleus esculentus, Droogmansia munamensis, Garcinia huillensis, Justicia insularis, Khaya nyasica, Oldfieldia dactylophylla, Senna petersiana, Senna singueana, Shirakiopsis elliptica, Steganotaenia araliacea, Uapaca robynsii, Vernonia excelsa, Brassica oleracea, Croton mubango, Vachellia karroo, Dichrostachys cinerea, Aloe vera, Senna occidentalis, Cassia abbreviata, Erythrina abyssinica, Salvia officinalis.* | |
| Gastrointestinal disorders | 30 | 0.181 | 1912 | 65.79 | *Elaeis guineensis, Cucumis melo, Harungana madagascariensis, Albizia adianthifolia, Uapaca kirkiana, Uapaca nitida, Hymenocardia acida, Bridelia atroviridis, Euphorbia heterophylla, Maprounea africana, Capsicum frutescens, Chamaemelum nobile, Carduus nyassanus, Bridelia ferruginea, Chromolaena odorata, Crassocephalum crepidioides, Euphorbia hirta, Ficus sur, Psidium guajava, Bridelia scleroneura, Cajanus cajan, Chenopodium opulifolium, Dalbergia boehmii, Diplorhynchus condylocarpon, Khaya anthotheca, Senna petersiana, Senna siamea, Terminalia mollis, Senna occidentalis, Erythrina abyssinica* | |
| Malaria | 29 | 0.175 | 1958 | 67.38 | *Julbernardia paniculata, Ochna schweinfurthiana, Gardenia ternifolia, Piliostigma thonningii, Xylopia aethiopica, Harungana madagascariensis, Crossopteryx febrifuga, Bidens pilosa, Dysphania ambrosioides, Carica papaya, Combretum molle, Lantana camara, Nauclea latifolia, Aloe buettneri, Baphia capparidifolia, Bobgunnia madagascariensis, Cajanus cajan, Crassocephalum montuosum, Dalbergia boehmii, Dalbergia nitidula, Heinsia crinita, Justicia insularis, Kalaharia uncinata, Psorospermum corymbiferum, Uapaca robynsii, Zanthoxylum chalybeum, Tetradenia riparia, Senna occidentalis, Erythrina abyssinica* | |
| Sexual Transmitted Infections | 23 | 0.139 | 1454 | 50.03 | *Albizia adianthifolia, Albizia antunesiana, Elaeis guineensis, Flueggea virosa, Uapaca nitida, Pseudolachnostylis maprouneifolia, Bridelia micrantha, Phyllanthus niruri, Gymnanthemum amygdalinum, Euphorbia hypericifolia, Euphorbia inaequilatera, Euphorbia tirucalli, Ziziphus mucronata, Euphorbia hirta, Ficus sur, Bobgunnia madagascariensis, Cleistanthus polystachyus, Ekebergia benguelensis, Euphorbia terracina, Garcinia huillensis, Hibiscus surattensis, Mucuna poggei, Senna singueana* | |
| Diarrhea | 23 | 0.139 | 679 | 23.37 | *Gardenia ternifolia, Piliostigma thonningii, Albizia adianthifolia, Uapaca sansibarica, Hymenocardia acida, Bridelia micrantha, Crossopteryx febrifuga, Musa × paradisiaca, Albizia gummifera, Aframomum melegueta, Ocimum gratissimum, Celosia trigyna, Psidium guajava, Nauclea latifolia, Albizia antunesiana, Monotes katangensis, Terminalia mollis, Triumfetta rhomboidea, Tetradenia riparia, Vachellia karroo, Psorospermum febrifugum, Erythrina abyssinica, Salvia officinalis* | |
| Fever | 19 | 0.114 | 1978 | 68.07 | *Entada africana, Jatropha curcas, Crossopteryx febrifuga, Bidens pilosa, Gymnanthemum amygdalinum, Pterocarpus angolensis, Chamaemelum nobile, Lantana camara, Nauclea latifolia, Diplorhynchus condylocarpon, Heinsia crinita, Jacobaea maritima, Monotes africanus, Monotes katangensis, Nicandra physalodes, Senna siamea, Zanha africana, Tetradenia riparia, Parinari curatellifolia, Psorospermum febrifugum, Ziziphus abyssinica.* | |
| Dysentery | 17 | 0.102 | 156 | 5.37 | *Bobgunnia madagascariensis, Bridelia ferruginea, Combretum molle, Croton mubango, Ekebergia benguelensis, Euphorbia hirta, Ficus thonningii, Hymenocardia acida, Musa × paradisiaca, Nauclea latifolia, Phyllanthus muellerianus, Psidium guajava, Terminalia mollis, Uapaca kirkiana, Uapaca pilosa, Vachellia karroo, Xylopia aethiopica.* | |
| Constipation | 15 | 0.090 | 176 | 6.06 | *Aframomum angustifolium, Bridelia ferruginea, Caladium bicolor, Combretum molle, Cucumis melo, Eleusine indica, Euphorbia heterophylla, Ficus thonningii, Gymnanthemum amygdalinum, Phyllanthus muellerianus, Securidaca longepedunculata, Senna occidentalis, Shirakiopsis elliptica, Steganotaenia araliacea, Uapaca pilosa.* | |
| Jaundice | 14 | 0.084 | 189 | 6.50 | *Acalypha chirindica, Albizia gummifera, Annona senegalensis, Cleistanthus polystachyus, Diospyros mespiliformis, Euphorbia hirta, Garcinia huillensis, Khaya nyasica, Oldfieldia dactylophylla, Penianthus longifolius, Phyllanthus niruri, Senna siamea, Senna singueana, Shirakiopsis elliptica.* | |
| Rheumatism | 13 | 0.078 | 112 | 3.85 | *Anonidium mannii, Curcuma longa, Cyanthillium cinereum, Dichrostachys cinerea, Erigeron sumatrensis, Flueggea virosa, Gardenia ternifolia, Lantana camara, Ricinus communis, Salvia officinalis, Senna singueana, Smilax anceps, Zanha africana.* | |
| Cough | 12 | 0.072 | 114 | 3.92 | *Antidesma venosum, Bidens pilosa, Curcuma longa, Euphorbia hirta, Gardenia ternifolia, Gymnanthemum amygdalinum, Maprounea africana, Markhamia lutea, Syzygium guineense, Tetradenia riparia, Xylopia aethiopica, Zanthoxylum chalybeum* | |
| Arterial hypertension | 11 | 0.066 | 108 | 3.72 | *Allium sativum, Antidesma venosum, Bidens pilosa, Curcuma longa, Euphorbia hirta, Gymnanthemum amygdalinum, Maprounea africana, Markhamia lutea, Syzygium guineense, Tetradenia riparia, Xylopia aethiopica.* |  |
| Headaches | 10 | 0,060 | 172 | 5,92 | *Aframomum alboviolaceum, Albizia gummifera, Combretum molle, Erigeron sumatrensis, Manihot esculenta, Maprounea africana, Markhamia lutea, Securidaca longepedunculata, Tetradenia riparia, Zanha africana.* | |
| Cancer | 10 | 0.060 | 145 | 4.99 | *Aloe vera, Brassica oleracea, Bridelia atroviridis, Carica papaya, Dalbergia boehmii, Gardenia ternifolia, Lantana camara, Ocimum gratissimum, Parinari curatellifolia, Pseudolachnostylis maprouneifolia* | |
| Gastritis | 10 | 0.060 | 109 | 3.75 | *Abelmoschus esculentus, Aframomum melegueta, Antidesma venosum, Combretum molle, Dysphania ambrosioides, Ekebergia benguelensis, Entada africana, Euphorbia hirta, Sterculia quinqueloba, Tetradenia riparia.* | |
| Gingivitis | 8 | 0.048 | 67 | 2.31 | *Acmella caulirhiza, Bridelia micrantha, Cajanus cajan, Cassia abbreviata, Dalbergia nitidula, Piliostigma thonningii, Tetradenia riparia, Ziziphus mucronata.* | |
| Hepatitis | 8 | 0.048 | 51 | 1.75 | *Abelmoschus esculentus, Allium sativum, Curcuma longa, Dysphania ambrosioides, Entada africana, Gardenia ternifolia, Imperata cylindrica, Uapaca robynsii.* | |
| Sexual dysfunction | 7 | 0.042 | 42 | 1.45 | *Flueggea virosa, Heinsia crinita, Khaya anthotheca, Moringa oleifera, Pterocarpus angolensis, Vachellia karroo, Ziziphus mucronata.* | |
| Pneumonia | 7 | 0.042 | 42 | 1.45 | *Acalypha psilostachya, Euphorbia hirta, Euphorbia hypericifolia, Garcinia huillensis, Khaya anthotheca, Parinari curatellifolia, Zanha africana.* | |
| Tumor | 6 | 0.036 | 48 | 1.65 | *Crassocephalum crepidioides, Euphorbia hirta, Lantana camara, Salvia officinalis, Triumfetta rhomboidea, Uapaca acuminata.* | |
| Peptic ulcer disease | 6 | 0.036 | 39 | 1.34 | *Dalbergia boehmii, Ficus sur, Imperata cylindrica, Lantana camara, Phyllanthus ovalifolius, Triumfetta rhomboidea* | |
| Human Immunodeficiency Virus | 6 | 0.036 | 34 | 1.17 | *Cassia abbreviata, Erythrina abyssinica, Euphorbia hypericifolia, Hypoestes triflora, Imperata cylindrica, Monotes africanus.* | |
| Bronchitis | 5 | 0.030 | 30 | 1.03 | *Acalypha paniculata, Acalypha petiolaris, Euphorbia hirta, Euphorbia hypericifolia, Musa × paradisiaca* | |
| Hemorrhoids | 5 | 0.030 | 27 | 0.93 | *Acalypha paniculata, Acalypha petiolaris, Euphorbia hirta, Euphorbia hypericifolia, Musa × paradisiaca* | |
| Intestinal worms | 5 | 0.030 | 26 | 0.89 | *Abelmoschus esculentus, Celosia trigyna, Cucumis sativus, Diospyros mespiliformis, Euphorbia hirta.* | |
| Tuberculosis | 4 | 0.024 | 31 | 1.07 | *Annona senegalensis, Crossopteryx febrifuga, Xylopia aethiopica, Zanthoxylum chalybeum* | |
| Sickle cell disease | 4 | 0.024 | 26 | 0.89 | *Annona senegalensis, Crossopteryx febrifuga, Xylopia aethiopica, Zanthoxylum chalybeum* | |
| Asthma | 4 | 0.024 | 25 | 0.86 | *Euphorbia hirta, Gardenia ternifolia, Lantana camara, Syzygium guineense* | |
| Anemia | 4 | 0.024 | 24 | 0.83 | *Cucumis melo, Harungana madagascariensis, Hypoestes triflora, Ocimum gratissimum.* | |
| Arthritis | 4 | 0.024 | 21 | 0.72 | *Bridelia ferruginea, Bridelia scleroneura, Paullinia pinnata, Piliostigma thonningii* | |
| Ascites | 3 | 0.018 | 17 | 0.58 | *Annona senegalensis, Crossopteryx febrifuga, Xylopia aethiopica, Zanthoxylum chalybeum* | |
| Cataracts | 3 | 0.018 | 15 | 0.52 | *Albertisia villosa, Entada africana, Lantana camara* | |
| Schistosomiasis | 3 | 0.018 | 13 | 0.45 | *Bobgunnia madagascariensis, Mucuna poggei, Pseudolachnostylis maprouneifolia* | |
| Leprosy | 3 | 0.018 | 12 | 0.41 | *Centella asiatica, Cucumis melo, Gardenia ternifolia* | |
| Dysmenorrhea | 3 | 0.018 | 11 | 0.38 | *Chamaemelum nobile, Erigeron sumatrensis, Lantana camara* | |
| Respiratory infections | 3 | 0.018 | 10 | 0.34 | *Commelina diffusa, Euphorbia terracina, Tetradenia riparia* | |
| Typhoid fever | 3 | 0.018 | 9 | 0.31 | *Bidens pilosa, Monotes katangensis, Senna siamea* | |
| Osteoarthritis | 2 | 0.012 | 14 | 0.48 | *Aframomum angustifolium, Ageratum conyzoides* | |
| Asthenias | 2 | 0.012 | 11 | 0.38 | *Chenopodium opulifolium, Chromolaena odorata* | |
| Urinary tract infections | 2 | 0.012 | 11 | 0.38 | *Commelina diffusa, Anisophyllea boehmii* | |
| Convulsion | 2 | 0.012 | 10 | 0.34 | *Caladium bicolor, Markhamia lutea* | |
| Insomnia | 2 | 0.012 | 10 | 0.34 | *Chamaemelum nobile, Ricinus communis* | |
| Gout | 2 | 0.012 | 9 | 0.31 | *Erigeron sumatrensis, Salvia officinalis* | |
| Snake bite | 2 | 0.012 | 8 | 0.28 | *Diplorhynchus condylocarpon, Flueggea virosa* | |
| Sinusitis | 2 | 0.012 | 6 | 0.21 | *Curcuma longa, Luffa aegyptiaca* | |
| Uretritis | 2 | 0,012 | 4 | 0.14 | *Bridelia atroviridis, Hibiscus surattensis* | |
| Anorexia | 1 | 0.006 | 7 | 0.24 | *Curcuma longa* | |
| Kidney stones | 1 | 0.006 | 6 | 0.21 | *Cucumis melo* | |
| Herpes | 1 | 0.006 | 6 | 0.21 | *Memecylon flavovirens* | |
| Epilepsy | 1 | 0.006 | 5 | 0.17 | *Ekebergia benguelensis* | |
| Anxiety | 1 | 0.006 | 5 | 0.17 | *Gardenia imperialis* | |
| Dyspnea | 1 | 0.006 | 5 | 0.17 | *Ageratum conyzoides* | |
| Meningitis | 1 | 0.006 | 5 | 0.17 | *Entada abyssinica* | |
| Weight loss | 1 | 0.006 | 4 | 0.14 | *Croton mubango* | |
| Amoeba | 1 | 0.006 | 4 | 0.14 | *Euphorbia hirta* | |
| Eczema | 1 | 0.006 | 4 | 0.14 | *Lantana camara* | |
| Obesity | 1 | 0.006 | 4 | 0.14 | *Brassica oleracea* | |
| Helminthiasis | 1 | 0.006 | 3 | 0.10 | *Anisophyllea boehmii* | |
| Measles | 1 | 0.006 | 3 | 0.10 | *Lantana camara* | |
| Amenorrhea | 1 | 0.006 | 3 | 0.10 | *Ziziphus abyssinica* | |
| Psoriasis | 1 | 0.006 | 3 | 0.10 | *Centella asiatica* | |
| Tooth decay | 1 | 0.006 | 2 | 0.07 | *Gardenia ternifolia* | |
| Angina | 1 | 0.006 | 2 | 0.07 | *Tetradenia riparia* | |
| Uterine fibroids | 1 | 0.006 | 2 | 0.07 | *Xylopia aethiopica* | |
| Flatulence | 1 | 0.006 | 2 | 0.07 | *Cucumis melo* | |
| Impetigo | 1 | 0.006 | 2 | 0.07 | *Kigelia africana* | |
| Eye infections | 1 | 0.006 | 2 | 0.07 | *Euphorbia inaequilatera* | |
| Facial paralysis | 1 | 0.006 | 2 | 0.07 | *Caladium bicolor* | |
| Varicella | 1 | 0.006 | 2 | 0.07 | *Albizia antunesiana* | |
| Vitiligo | 1 | 0.006 | 2 | 0.07 | *Aloe buettneri* | |

***Legend*** - NC= number of times the pathology was cited; nt= number of taxa involved in the management of the pathology cited; MCI= Medical Capability Index. The Medical Capability Index (MCI) refers to a community's potential to manage disease.
